# Gut microbiota regulates hepatic ketogenesis and lipid accumulation in ketogenic diet-induced hyperketonemia by disrupting bile acid metabolism

**DOI:** 10.1080/19490976.2025.2496437

**Published:** 2025-04-23

**Authors:** Zhengzhong Luo, Yixin Huang, Kang Yong, Dan Wu, Linfeng Zheng, Xueping Yao, Liuhong Shen, Shumin Yu, Baoning Wang, Suizhong Cao

**Affiliations:** a College of Veterinary Medicine, Sichuan Agricultural University, Chengdu, China; b Key Laboratory of Animal Disease and Human Health of Sichuan Province, Chengdu, China; c College of Animal Science and Technology, Chongqing Three Gorges Vocational College, Chongqing, China; d West China School of Basic Medical Sciences and Forensic Medicine, Sichuan University, Chengdu, China

**Keywords:** Ketogenic diet, hyperketonemia, gut microbiota, ketogenesis, bile acids, *Clostridium perfringens*

## Abstract

The ketogenic diet (KD) induces prolonged hyperketonemia, characterized by elevated circulating level of β-hydroxybutyrate. However, the KD can negatively affect host metabolic health by altering the gut microbial community. Despite this, the regulatory effect of the gut microbiota on hepatic ketogenesis and triacylglycerol (TAG) accumulation during a KD remains poorly understood. Here, we hypothesized that the commensal bacterium regulates hepatic lipid metabolism in association with KD-induced hyperketonemia. The KD disrupts the remodeling of the gut microbiota following antibiotic-induced depletion. The capacity for ketogenesis and the severity of TAG accumulation in the liver closely correlated with changes in the gut microbial composition and the up-regulation of hepatic farnesoid X receptor (*FXR*), peroxisome proliferator-activated receptor alpha (*PPARα*), and diacylglycerol O-acyltransferase 2 (*DGAT2*), which were modulated by bile acid metabolism through the gut-liver axis. The commensal bacterium *Clostridium perfringens* type A is particularly implicated in prolonged hyperketonemia, exacerbating hepatic ketogenesis and steatosis by disrupting secondary bile acid metabolism. The increased conversion of deoxycholic acid to 12-ketolithocholic acid represents a critical microbial pathway during *C. perfringens* colonization. These findings illuminate the adverse effects of the gut microbiota on hepatic adaptation to a KD and highlight the regulatory role of *C. perfringens* in ketonic states.

## Introduction

Physiological ketosis or hyperketonemia is characterized by a significant increase in circulating ketone bodies, particularly, β-hydroxybutyrate (BHB), which is a major molecule associated with metabolism.^1,2^ Under negative energy balance or glucose deficiency, the breakdown of fatty acids is metabolized in the liver to produce BHB through ketogenesis, serving as an alternative fuel for extrahepatic tissues.^3^ Current perspectives suggest that BHB functions as a metabolic messenger, positively regulating mitochondrial metabolic homeostasis, neuroprotection and immune systems.^2,4,5^ Ketogenic dysfunction in the liver is closely linked to the development of metabolic disorders.^6^ In ruminants, prolonged hyperketonemia is recognized as a contributor to the occurrence of disease.^7^ During hepatic ketogenesis, mitochondrial hydroxy-methylglutaryl CoA synthetase 2 (HMGCS2), a key rate-limiting enzyme, condenses acetyl-CoA with acetoacetyl-CoA to form 3-hydroxy-3-methylglutaryls-CoA, which is further metabolized to acetoacetate and BHB. Caloric restriction, fasting, and ketogenic diet (KD) are considered effective methods of enhancing blood BHB level.^1,8,9^ Generally, hepatic glycogen depletion following long-term fasting leads to a rapid increase in blood BHB level, which subsequently decline upon refeeding.^10,11^ In contrast, the KD is a very low-carbohydrate and high-fat diet, which induces prolonged hyperketonemia during glucose deficiency.^12^ Nutritional trials indicate that following a KD can result in significant weight loss and improvements in metabolic disorders associated with obesity or fatty liver disease.^13–15^ However, some studies report that the KD may negatively affect metabolic health, contributing to hepatic insulin resistance and lipid accumulation.^16–19^ Additionally, the KD aggravates dysfunction of the intestinal barrier by promoting the colonization of pathogenic taxa, as reported in a previous study.^20^ So, understanding the negative effects of hyperketonemia, particularly regarding its association with metabolic disorders related to the KD, is essential.

Dietary patterns substantially influence the metabolic health of the host by manipulating the gut microbiome.^21^ Among various dietary patterns, alterations in gut microbial composition due to KD consumption are closely related to changes in metabolic phenotypes, such as insulin resistance and hepatic lipid accumulation.^17,22^ The KD has alters the relative abundance of certain intestinal commensal bacteria, particularly affecting *Akkermansia, Bifidobacterium, Escherichia*, and *Clostridium*. ^4,23,24^ The production of ketone bodies in the host following a KD, mainly BHB considerably influences the composition of gut microbiota and selectively inhibits the growth of *Bifidobacteria* in the intestine.^25,26^ In contrast to findings that the KD alters the composition of gut microbiota, its effects on the homeostasis and remodeling of gut microbial community are unclear. Conversely, gut microbes can also influence lipid metabolism during consumption of a high-fat diet.^27,28^ For instance, the commensal bacterium *Clostridium sporogenes* enhances the colonization of *Clostridium* species, subsequently exacerbating fat accumulation by increasing energy absorption.^29^ Although the interaction between diet and gut microbiota has been documented,^21^ the regulatory effect of commensal bacteria on lipid metabolism during KD-induced hyperketonemia remains poorly understood. Metabolites derived from gut microbiota participates in the regulation of host health across various dietary patterns.^21,30^ The alteration in gut microbial-dependent bile acid metabolic pattern associated with KD consumption influences the body weight loss and fasting glucose level.^31^ Bile acids are important signaling molecules that regulate physiological metabolic responses in the host through bidirectional communication along the gut-liver axis.^32,33^ Our prior study confirmed the association among bile acids, gut microbiota, and the development of hyperketonemia.^34^ However, whether gut microbes regulate the ketogenic capacity and lipid metabolism in hyperketonemia by disrupting bile acids is unclear.

Therefore, this study aimed to elucidate the regulatory mechanism of gut microbiota in hepatic ketogenesis and lipid synthesis during KD-induced hyperketonemia. First, we analyzed dynamic differences in ketogenesis during two weeks of a KD consumption and its association with bile acid metabolism using antibiotic-induced microbiome depletion. Second, we investigated the relationship between gut microbiota composition and ketogenic capacity by transplanting faecal microbiota from donors with varying blood BHB levels. Finally, we identified key gut commensal bacteria based on different treatments during KD consumption and explored the regulatory pathways of these microbes in maintaining hepatic metabolic homeostasis. These study findings could provide new insights into the adverse effects of the KD on metabolic health.

## Materials and methods

### Animals and management

Female C57BL/6J mice (aged 6 weeks; 16–17 g) were purchased from SPF Biotechnology Co., Ltd. (Beijing, China). The mice were acclimated for one week to standard environmental conditions (temperature: 23  ±  3°C; humidity: 52  ±  8 %) on a 12:12 h light-dark cycle before the dietary intervention. All animal procedures were conducted in accordance with the guidelines and approved by the Animal Care and Use Committee of Sichuan Agricultural University (approval ID: 20220163 and 20230047). Two dietary treatments were administered to the mice: a KD (No. XTKD01, comprising 89.99 % fat and 10 % protein, with 0.01 % carbohydrates, on a kilocalorie basis) and a control normal chow of KD (No. XTKDCON, containing 10.06 % fat, 79.94 % carbohydrates, and 10 % protein). Both diets were purchased from Jiangsu Xietong Pharmaceutical Bio-engineering Co., Ltd. (Nanjing, China). Supplementary Table S1 details the ingredient composition and nutritional level of the diets.

### Trials of treatment in mice

#### Trial 1. Antibiotics (Abx) and ketogenic dietary treatment

Fifty C57BL/6J mice were randomly assigned to four groups: phosphate-buffered saline (PBS) pre-treatment with a normal diet (PND; *n* = 10), PBS with pre-treatment a KD (PKD; *n* = 15), Abx pre-treatment with a normal diet (AND; *n* = 10), and Abx pre-treatment with a KD (AKD, *n* = 15). Four types of antibiotics were dissolved in phosphate-buffered saline (PBS) to prepare the Abx cocktail. The cocktail (Beyotime Biotechnology, China) comprised 200 mg/kg ampicillin, 200 mg/kg neomycin, 200 mg/kg metronidazole, and 100 mg/kg vancomycin, based on the body weight of the mice.^35^ The AND and AKD groups were intragastrically administered an Abx cocktail once daily for 7 consecutive days to deplete the gut microbiota.^36^ Conversely, the PND and PKD groups were administered an equal volume of PBS intragastrically once daily for the same duration. After fasting for 24 h, the PKD and AKD groups mice were fed a KD for two consecutive weeks, while the PND and AND groups mice were fed a control diet, as described in Supplementary Figure S1a. All the mice had ad libitum access to water and food, and body weight were measured throughout the experimental period. Blood samples collected from the tail vein on days 2, 4, 8, and 14 during ketogenic or control dietary treatment and after fasting, were analyzed for BHB and glucose levels using blood ketone meters (Abbott Laboratories Co., Abbott Park, IL, USA). Following the consumption of KD, the samples of mice blood, liver, and faeces were collected from all groups. Faecal microbiota analysis were performed using full-length 16S rRNA amplicon sequencing.

#### Trial 2. Faecal microbiota transplantation

Faeces donors consisted of 10 healthy dairy cows (HE, blood BHB = 0.9  ±  0.18 mmol/L) and 10 hyperketonemic dairy cows (HYK, blood BHB = 3.5  ±  0.79 mmol/L). Detailed information about the dairy cows is available in our previous report.^34^ On the day of faecal microbiota transplantation (FMT), 5 g of faecal samples from each cow within the same group were mixed and suspended in sterile PBS at a concentration of 50 mg faeces/mL.^4,35^ The resulting supernatant was collected after centrifugation at 100 × *g* for 2 min at 4°C. Twenty-four C57BL/6J mice were randomly assigned to the FMT-HE (*n* = 12) and FMT-HYK (*n* = 12) groups. All the mice were administered an Abx cocktail through oral gavage once daily for 7 consecutive days. Following one day without the Abx cocktail, the female adult mice were colonized with 300 μL of supernatant obtained from the different cow donors for 10 consecutive days.^37,38^ After acclimating for 2 days, the mice were fasted for 24 h and then fed a KD for two consecutive weeks, with *ad libitum* access to food and water. The levels of BHB and glucose in blood were measured during weeks 1 and 2 of a KD and after fasting. The samples were collected in the same manner as in Trial 1.

#### Trial 3. Commensal bacteria cultivation and intervention

Fresh faecal samples from healthy C57BL/6J mice were collected and added to the reinforced *Clostridial* media. After being cultured anaerobically for 4 h at 41°C, the suspension culture was inoculated onto tryptose sulfite cycloserine agar (Merck KGaA, Darmstadt, Germany) medium and incubated anaerobically for 24 h at 41°C. Subsequently, the black colonies were inoculated into a sheep blood agar (Thermo Fisher Scientific Inc., USA) medium and anaerobically cultured for 24 h at 41°C. Individual colonies were selected and inoculated into a *Clostridium* growth medium, followed by an 8 h incubation at 41°C. After three rounds of purification on the sheep blood agar medium, the morphology and identification of *C. perfringens* were analyzed using Gram staining and PCR methods; the reaction condition of the PCR are outlined in Supplementary Table S2. The toxin typing of *C. perfringens*, including alpha, beta, epsilon, and iota toxins, was conducted using specific primers, with the PCR primer information provided in Supplementary Table S3. Based on the types of toxin, the *C. perfringens* toxinotype was identified.^39^ Sixteen female C57BL/6J mice were randomly assigned to two groups: *C. perfringens* (*n* = 8) and vehicle (*n* = 8). *C. perfringens* was dissolved in sterile PBS to achieve a final concentration of 1  ×  10^8^ CFU per mL and administered orally at 200 μL once daily for 14 consecutive days.^29,40^ The mice in the vehicle group were administered the same volume of PBS. All the mice were fed a KD for 14 consecutive days. The detection of BHB and glucose levels in blood, as well as the collection of samples, were conducted in the same manner as in Trial 2. Liver and colonic contents were collected, and then metabolomics analysis was conducted using liquid chromatography-tandem mass spectrometry.

### Serum and liver markers determination

The serum levels of non-esterified fatty acids (NEFA), triacylglycerol (TAG), total cholesterol (TCHO), and insulin were measured using a commercial kit from the Nanjing Jiancheng Bioengineering Institute (Nanjing, China), following the instructions of the manufacturer. To prepare the tissue homogenate, 100 mg of liver samples were homogenized with 900 μL of absolute ethyl alcohol. After centrifugation at 2500 × *g* for 10 min at 4°C, the resulting supernatant was collected, and levels of NEFA, TAG, and THCO were subsequently determined according to the instructions of the manufacturer.

### Histological analysis

All the liver samples were fixed in 4 % paraformaldehyde and stored at 25°C. The tissue was then embedded and sectioned into 5 µm slices using a Leica RM2235 microtome (Leica Biosystems, Wetzlar, Germany). Sections were stained with hematoxylin and eosin (H&E) and examined under an optical microscope (DM500; Leica Biosystems, Wetzlar, Germany). Additionally, the fixed tissue was dehydrated in 30 % sucrose, embedded in Tissue-Tek® O.C.T. compound (Sakura Finetek, USA), and sliced into 7 µm sections. Histopathological scoring of the liver was conducted according to established scoring systems for nonalcoholic fatty liver disease.^41^ The positive area of oil staining in the liver was quantified and analyzed automatically using Image-Pro Plus software (v 7.0, Rockville, Maryland, USA).

### Full-length 16S rRNA amplicon sequencing

In Trial 1 and 2, following the consumption of the KD, the faeces samples of mice were collected from all group and microbiota analysis were performed. Genomic DNA of faeces was extracted using a commercial kit (DP712, TIANGEN Biotech Co., Ltd, Beijing, China). The full-length 16S rRNA gene was amplified with the primer pairs 27F: 5′-AGRGTTYGATYMTGGCTCAG-3′ and 1492 R: 5′-RGYTACCTTGTTACGACTT-3′.^42^ PCR amplification was performed in a 20 μL reaction volume under the following thermal conditions: initial denaturation at 95°C for 2 min, followed by 25 cycles of denaturation at 95°C for 30 s, primer annealing at 60°C for 30 s, and extension at 72°C for 1 min. A final extension was performed at 72°C for 5 min. The amplificated products were purified, before being quantified using a QuantiFluor^TM^-ST Fluorometer (Promega Biotech Co., Ltd, Madison, WI, USA). Subsequently, the 16S rRNA gene amplicon was sequenced using the PacBio Sequel II platform (San Diego, CA, USA). The sequencing data was spliced, filtered, and denoised as described in our previous study.^34^ Taxonomic classification of the amplicon sequence variants (ASVs) was performed using the SILVA v138.1 database (https://www.arb-silva.de/documentation/release-1381). Functional prediction of the gut microbiota was conducted using a phylogenetic investigation of communities by reconstruction of unobserved states 2.0.^43^

### Bile acids profiling analysis

A 25 μL serum sample was combined with 120 μL of ice-cold methanol containing partial internal standards in a 96-well plate. The mixture was vortexed for 5 min and centrifuged at 4000 × *g* for 30 min at 4°C, after which the resulting supernatant was collected. Following derivatization, the supernatant was mixed with 10 μL of internal standards, and thereafter bile acids profiling analysis was performed using an ultra-performance liquid chromatography coupled to a tandem mass spectrometry system (ACQUITY UPLC-Xevo TQ-S, Waters Corp., Milford, MA, USA). The chromatographic and mass spectrometry conditions were based on published methods.^44,45^ Metabolomics data were processed using the TMBQ software to integrate peaks, calibrate, and quantify the bile acids.^46^

### Metabolomics profiling analysis

A 25 mg sample of the liver tissue or colonic contents was added into 500 μL of a methanol-acetonitrile-water solution (v/v/v = 2:2:1) containing isotope-labeled internal standards and then homogenized at 4°C and 35 hz for 4 min. The mixtures were then placed in an ice bath and sonicated for 5 min, and this process was conducted in triplicate. The supernatant was collected after centrifugation at 4°C and 13,800 × *g* for 15 min. Supernatants were analyzed using liquid chromatography-tandem mass spectrometry. Chromatographic separations were performed with an ultra-high performance liquid chromatograph (Vanquish, Thermo Fisher Scientific, USA) equipped with a BEH Amide column (2.1 mm  ×  100 mm, 2.6 μm; Water Corporation, USA). The chromatographic conditions included the following: an injection volume of 2 μL, an auto-sampler temperature of 4°C, mobile phase A consisting of 25 mmol/L ammonium acetate and 25 mmol/L ammonia, and mobile phase B being acetonitrile. Subsequently, the samples were analyzed using an Orbitrap Exploris™ 120 mass spectrometer (Thermo Fisher Scientific, USA). Mass spectrometry detection parameters included a capillary temperature of 320°C, a full MS scan resolution of 60,000, an MS/MS scan resolution of 15,000, and spray voltages of 3.8 kV in positive ion mode and  − 3.4 kV in negative ion mode. Raw data were converted to the mzXML format using ProteoWizard software, and retention time, peak alignment, and peak area were extracted using XCMS software. Metabolite identification was performed by matching with the BiotreeDB (v3.0) database, following MSI level 1 standards.^47^ After filtering and filling in missing values, the metabolomic feature data were normalized using an adjusted internal standard. Principal component analysis (PCA) and orthogonal partial least squares discriminant analysis (OPLS-DA) of the feature data were conducted using SIMCA software. Differential analyses of the metabolites were determined based on variable importance in projection (VIP >1) from OPLS-DA, with the p-value calculated using the Student’s *t*-test.

### Liver RNA isolation and real-time qPCR analysis

After homogenization at 4°C, total RNA was extracted from the liver tissue using a commercial kit (DP451, TIANGEN Biotech Co., Ltd, Beijing, China). RNA quality and concentration were assessed according to the MIQE guidelines.^48^ Reverse transcription of total RNA was performed using a FastKing RT Kit (TIANGEN Biotech Co., Ltd). Quantitative analysis of the target genes was performed with a Real-Time qPCR system (CFX Opus 96, Bio-Rad Laboratories, Inc., California, USA) using the FastReal SYBR Green Master Mix (TIANGEN Biotech Co., Ltd). Supplementary Table S4 presents the primer sequences for the targeted genes. Relative gene expression levels were quantified and normalized to the internal reference gene β-actin using the ΔΔCT analysis method.

### Statistical analysis

All plots were generated using GraphPad 10.0 (GraphPad Software, San Diego, USA) and R software (version 4.3.1). Differential analysis among multiple groups was performed using ANOVA or the Kruskal – Wallis test, as appropriate, in GraphPad 10.0. The p-value for multiple hypothesis testing was adjusted using the Benjamini – Hochberg method. For comparisons between two groups, differential analysis utilized a two-tailed unpaired *t*-test or the Mann – Whitney U test, as appropriate. Alpha and beta diversities of the microbial community were assessed using the R package ‘Picante’ (version 1.8.2) and ‘Vegan’ (version 2.6–6.1). Linear discriminant analysis effect size (LEfSe) was performed to identify biomarkers using OmicStudio tools (https://www.omicstudio.cn/tool/).^49^ The different patterns of the microbial co-occurrence network were analyzed using the R package ‘igraph’ (version 2.0.3), while network visualization and topology analysis were performed with the Gehpi software (version 0.10.1).

## Results

### A ketogenic diet increases circulating BHB level and liver TAG accumulation

**Figure 1.**
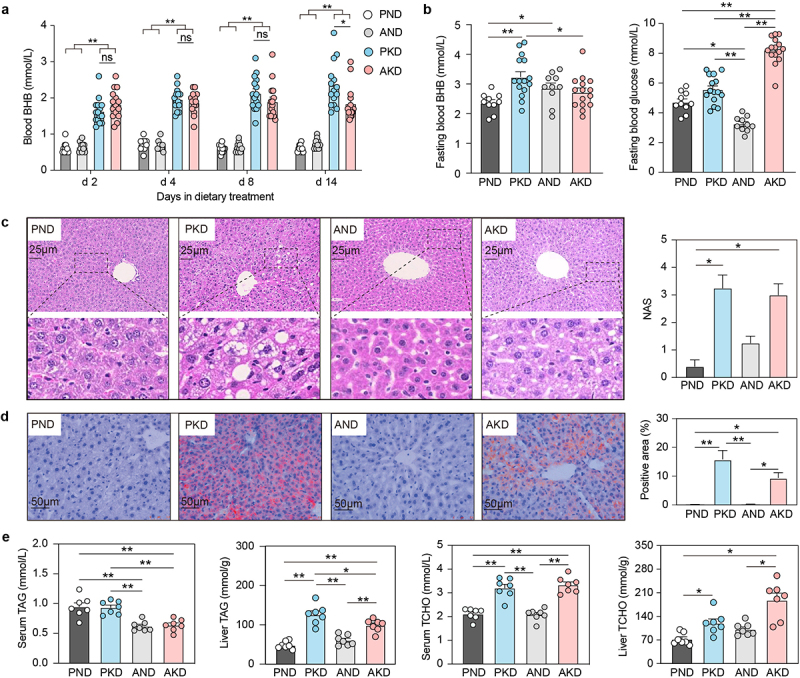
The ketogenic diet enhances ketogenesis and triacylglycerol(TAG) accumulation in female C57BL/6J mice. (a) Blood levels of beta-hydroxybutyrate (BHB) were compared among groups on days 2, 4, 8, and 14 following the ketogenic diet (PND and AND, n = 10 per group; PKD and AKD, n = 15 per group). (b) Fasting blood samples were analyzed to compare BHB and glucose levels among groups. (c) H&E staining (200×) of the liver was used to assess histopathology changes, with non‐alcoholic fatty liver disease activity scores (NAS) evaluated for each group (n = 7 per group). (d) Oil red O staining (400×) of the liver was performed to analyze the positive area among groups (n = 7 per group). (e) Changes in TAG and total cholesterol (TCHO) levels were measured across different treatments (n = 7 per group). Data are presented as mean ± SEM. **p < 0.01, *p < 0.05.

No notable differences in body weight were observed among the four groups during the pre-treatment and initial fasting periods (Supplementary Figure S1b). However, following fasting, mice on the KD exhibited significantly higher body weight than those on the control diet. Dynamic monitoring of circulating BHB level showed that the KD significantly increased blood BHB concentration, with significant differences observed between the PKD and AKD groups on day 14 of dietary treatment and fasting (Figure 1a,b). The glucose level in the AND group was lower than that in the PND group while fasting led to a significant increase in glucose levels in the AKD mice compared to those in the PKD mice (Figure 1b). The KD significantly increased TAG accumulation in the liver, while serum TAG level was reduced in the AKD group compared to those in the PKD group (Figure 1c–e). Additionally, TCHO levels in serum and liver were significantly elevated in the mice fed the KD.

### Ketogenic diet affects the composition and interaction of the gut microbial community

**Figure 2.**
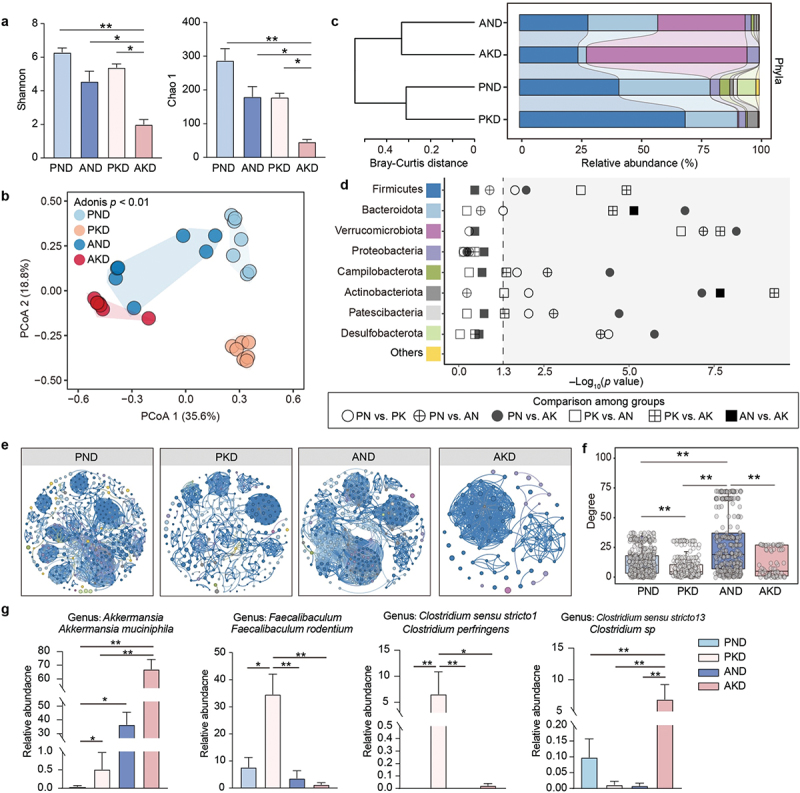
The ketogenic diet disrupts gut microbiota remodeling. (a) Alpha diversity of faecal microbiota in mice is compared among the different groups (n = 7 per group). (b) The principal coordinates analysis (PCoA) plot illustrates the differences in faecal microbiota community structure among the groups (n = 7 per group) based on the Bray–Curtis distance after 999 permutations. Adonis analysis was performed to compare the differences among the treatment groups. (c) The stacked bar plot presents the composition of faecal microbiota at the phylum level. (d) Relative abundance among the groups at the phylum level was compared using the Kruskal – Wallis test with the Benjamini – Hochberg adjustment. (e) The co-occurrence network analysis depicts associations among the ASV based on Spearman’s correlation coefficient (only |r| > 0.6 and p < 0.05 are shown). Nodes are color to represent different phylum taxa. (f) Box plot showing the topological degree differences in the co-occurrence network among the four groups. (g) Key bacteria are identified from the screened differential genera based on Kruskal – Wallis test and linear discriminant analysis effect size. Data are presented as mean ± SEM. **p < 0.01, *p < 0.05.

The Chao1 and Shannon diversities of gut microbial community was decreased in mice after consumption of the KD, with the lowest values observed in the AKD group (Figure 2a). Despite the varying dietary patterns, the diversity of microbiota was also reduced in mice treated with Abx depletion. In addition, both Abx and dietary treatments altered the gut microbiota structure, of which the structural heterogeneity of community was significantly altered in the PKD and AKD groups (Figure 2b). The composition of gut microbiota changed during Abx and dietary treatments. Notably, the relative abundance of Verrucomicrobiota was significantly higher in mice that pretreated with Abx (Figure 2c,d). In contrast to the PND group, mice in the PKD and AKD groups exhibited decreased relative abundances of Bacteroidota and Desulfobacterota. Furthermore, the relative abundance of Bacteroidetes and Actinobacteriota in the AND group was higher than that in the AKD group. These findings suggest that a KD impedes the remodeling of core bacteria after antibiotic treatment.

To examine microbial interactions, we conducted co-occurrence network analyses across the four groups based on significant correlations among ASVs. The co-occurrence networks revealed distinct clustering patterns of microbial interactions under Abx and dietary treatments (Figure 2e,f). In the PND group, the network consisted of 430 nodes (based on ASVs level) and 2,709 edges, resulting in an average degree of 12.6 (Supplementary Table S5). In contrast, the AND group included 310 nodes and 4,218 edges, yielding an average degree of 27.21, which is the highest among these group. The KD treatment reduced the number of nodes and edges within the network. Following Abx pre-treatment, the KD significantly reduced the diversity and complexity of the microbial network. The AKD group exhibited the lowest degree distribution, with 77 nodes and 439 edges, alongside the shortest average path length, as well as lower modularity and betweenness centrality than that of the other groups, highlighting a cohesive network structure. Various topological characteristics were utilized to assess intricate co-occurrence patterns. Besides the AKD group, the modularity index for the other three groups exceeded 0.4, indicating a modular network structure with limited interdependence among microorganisms.

The faeces core genera were significantly altered among the four groups, including *Akkermansia*, *Clostridium sensu stricto 1*, *Clostridium sensu stricto 13*, *Lactobacillus*, *Escherichia-Shigella*, *Bacteroides*, and *Faecalibaculum* (Supplementary Figure S2a). The LEfSe analysis showed *Akkermansia*, *Clostridium sensu stricto 1*, *Clostridium sensu stricto 13*, and *Faecalibaculum* as significant biomarkers in the dietary treatment (Supplementary Figure S2b). *Akkermansia muciniphila*, belonging to the *Akkermansia* genus, was increased in the mice with Abx pre-treatment, resulting in higher abundance in the AKD group (Figure 2g). The relative abundances of *Clostridium perfringens* and *Faecalibaculum rodentium* increased in mice fed a KD, with higher levels in the PKD group compared with those in the AKD group. Additionally, the relative abundance of *Clostridium sp*. significantly decreased in the PKD group compared with that in the AKD group.

### Ketogenic diet affects bile acid metabolism by manipulating the gut microbial function

**Figure 3.**
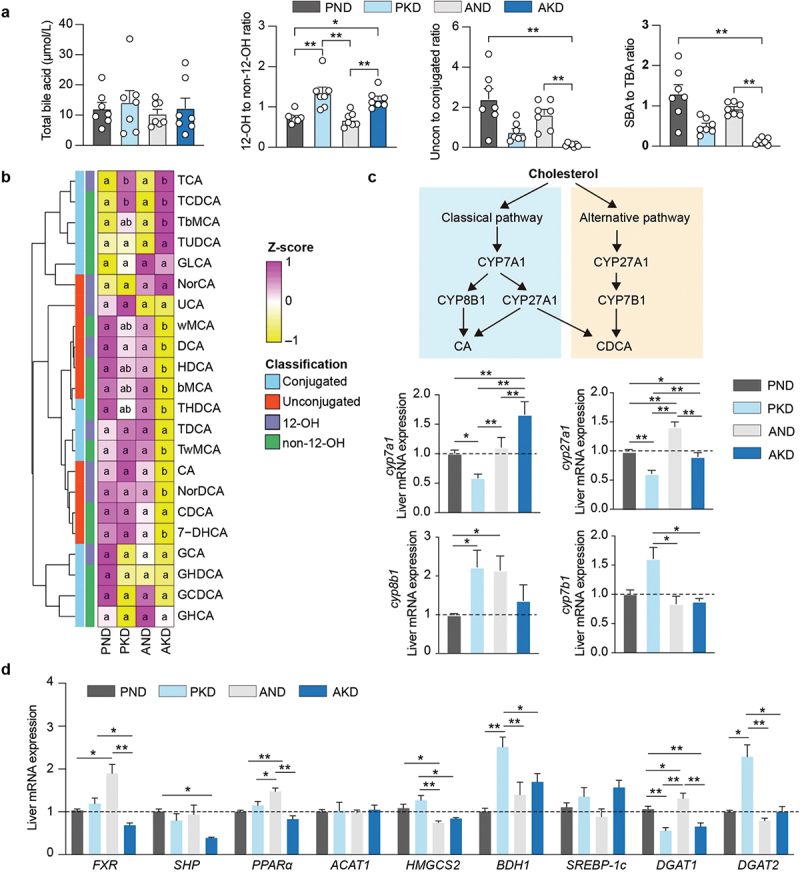
Effect of gut microbiota on bile acid metabolism and ketogenesis. (a) Total bile acids levels and composition ratios of bile acids in serum were compared across four groups (n = 7 per group). (b) The heatmap illustrates the relative change in individual bile acids in serum (n = 7 per group). Same lowercase letters on the same line for the same metabolite denote no significant difference, while different letters signify a significant difference. (c) Expression levels of key genes involved in the bile acid classic or alternative synthesis pathways were measured in the liver (n = 5  ~  6 replicates/treatment). (d) Relative mRNA expressions of key genes in lipogenesis and ketogenesis pathways were analyzed across different groups (n = 5  ~  6 replicates/treatment). Data are presented as mean ± SEM. **p < 0.01, *p < 0.05.

To investigate the relationship between the gut microbiota and glucolipid metabolism during KD consumption, we examined 12 pathways at the KEGG level 3. These pathways included glycolysis or gluconeogenesis, lipid metabolism, and secondary bile acid (SBA) biosynthesis (Supplementary Figure S3a). We observed a strong positive correlation (*r* = 0.61, *p* < 0.001) between circulating BHB level and SBA biosynthesis (Supplementary Figure S3b). Secondary bile acid biosynthesis was higher in the PKD group than in the AKD group (Supplementary Figure S3c). Following this, we conducted bile acid profiling for the different groups. Total serum bile acid levels did not differ significantly among the groups (Figure 3a). In mice fed a KD, the ratio of 12-OH to non-12-OH bile acids increased. Additionally, the ratios of unconjugated to conjugated bile acids and SBA to total bile acids were lowest in the AKD group. Among individual bile acids, TCA and TCDCA levels were higher in the PKD and AKD groups than those in the PND and AND groups (Figure 3b). Conversely, levels of some SBAs, such as TωMCA, ωMCA, THDCA, and TDCA, were lower in the AKD group than those in the other groups. We identified regulatory genes associated with bile acid metabolism in the liver samples based on classical and alternative pathways (Figure 3c). The relative expression of *CYP27A1* mRNA was downregulated in the PKD group compared to those in the control group. The expression of *CYP8B1* mRNA was higher in the PKD group than in the PND group. To further explore the relationship between bile acids, ketogenesis, and lipid synthesis, we assessed the relative expression of hepatic-targeted genes (Figure 3d). Regarding bile acid receptor expression, hepatic *FXR* mRNA was higher in the PKD group than in the AKD group but lower than in the AND group. Within the ketogenesis pathway, hepatic *HMGCS2* and *BDH1* were downregulated in the AKD group compared to those in the PKD group. Additionally, *DGAT2*, a key gene regulating TAG *de novo* synthesis, was significantly up-regulated in the PKD group.

### Gut microbiota contributes to hepatic ketogenesis and TAG accumulation during hyperketonemia

**Figure 4.**
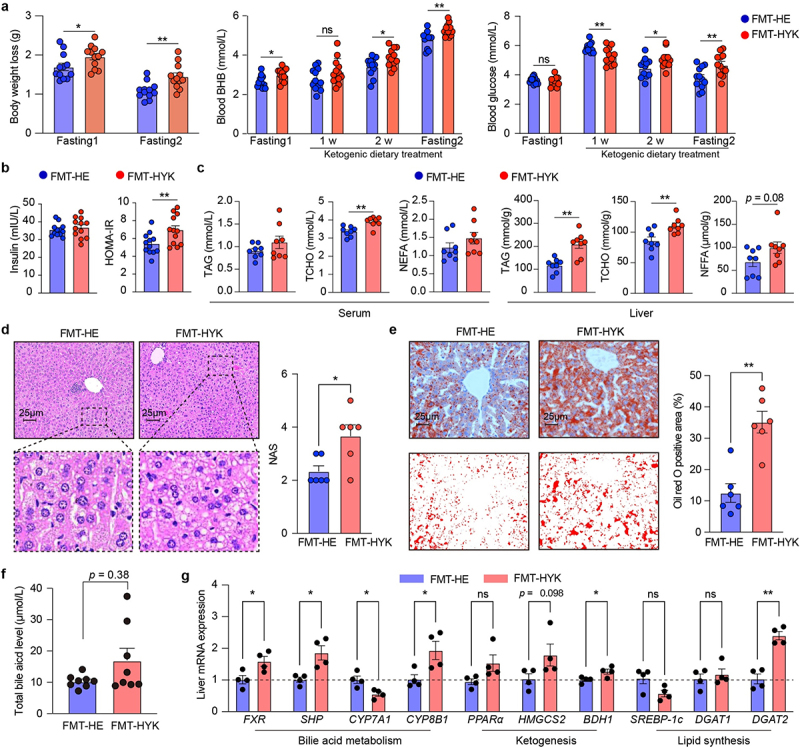
Alterations in clinical phenotypes and lipid metabolism of mice induced by differential gut microbiota composition following faecal microbiota transplantation (FMT) from various dairy cow donors. (a) Body weight loss was assessed during initial fasting after FMT treatment (Fasting1) and during subsequent fasting following KD treatment (Fasting2). Blood levels of BHB and glucose were measured during Fasting1, after 1 week of KD treatment, after 2 weeks of KD treatment, and during Fasting2. (b) The homeostatic model assessment of insulin resistance (HOMA-IR) was calculated based on glucose and insulin levels. (c) Serum and liver levels of triacylglycerol (TAG), total cholesterol (TCHO), and non-esterified fatty acids (NEFA) were measured during Fasting2 (n = 8 per condition). (d) Non-alcoholic fatty liver disease activity scores (NAS) were evaluated based on H&E staining (200×, n = 6 per condition). (e) Liver lipid accumulation was assessed using oil red O staining (400×, n = 6 per condition). (f) Serum total bile acid concentration was determined based on individual bile acid levels. (g) Relative mRNA expression of key genes involved in lipid metabolism was analyzed (n = 4 per condition). Data are presented as mean ± SEM. **p < 0.01, *p < 0.05.

To investigate the causality between the gut microbiota and ketogenesis, we performed FMT from differential donors inducing before long-term hyperketonemia (Supplementary Figure S4a). Following FMT treatment, no difference was observed in the body weight between the FMT-HE and FMT-HYK groups (Supplementary Figure S4b). However, during prolonged fasting, the FMT-HYK group exhibited significantly greater body weight loss and higher BHB levels than those in the FMT-HE group (Figure 4a). Following two weeks of KD intervention, the blood BHB and glucose levels increased in the FMT-HE group relative to that in the FMT-HYK group. Additionally, the HOMA-IR was higher in the FMT-HYK group than in the FMT-HE group (Figure 4b). Furthermore, liver TAG accumulation was greater in the FMT-HYK group; however, circulating TAG and NEFA levels did not differ between the two groups (Figure 4c-e). Although the bile acid pool size was no difference between both groups, the levels of HDCA, βUDCA, and 7-ketoLCA significantly increased in the FMT-HYK group (Figure 4f and Supplementary Figure S5). We also assessed the relative expression of bile acid-related genes in the liver. The expression of *FXR*, *SHP*, and *CYP8B1* was significantly up-regulated in the FMT-HYK group compared to that in the FMT-HE group (Figure 4g). Regarding the ketogenesis and lipid synthesis pathway, the expressions of *HMGCS2*, *BDH1*, and *DGAT2* were also up-regulated in the FMT-HYK group.

### Alteration in the gut microbial community associated with enhanced ketogenesis during hyperketonemia

**Figure 5.**
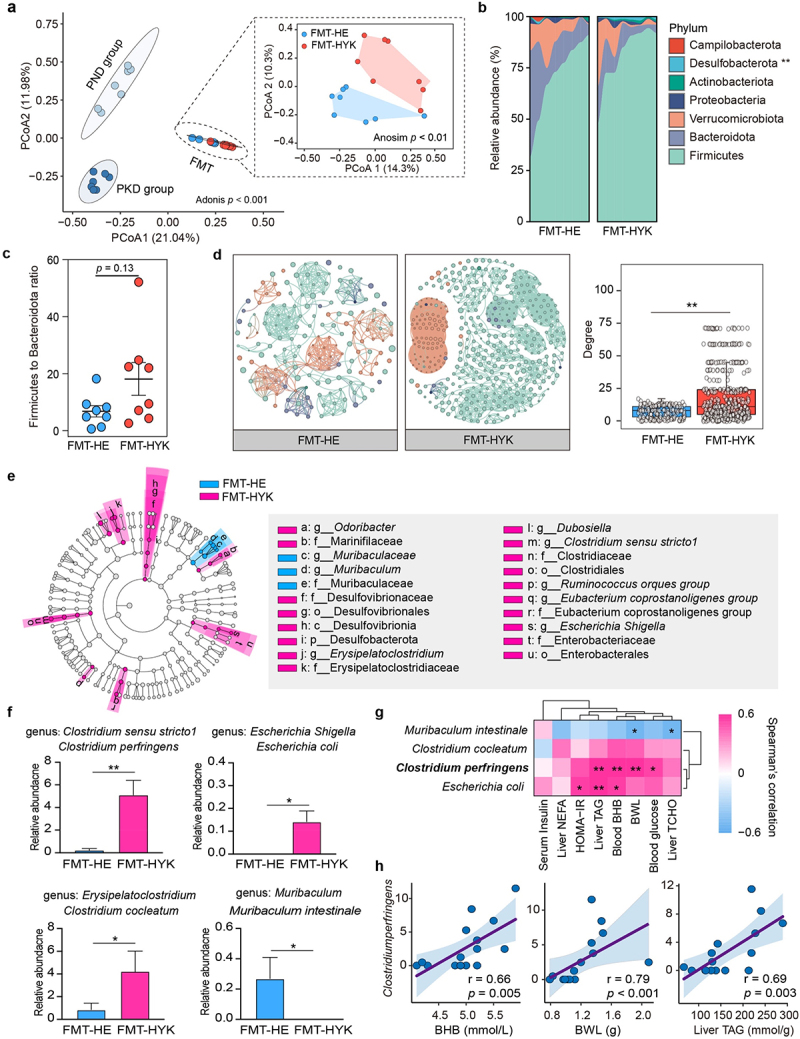
Alterations in faecal microbiota composition associated with hepatic ketogenesis and lipid accumulation during prolonged hyperketonemia. (a) Principal coordinates analysis (PCoA) shows the dissimilarity in faecal microbiota between the recipient and faecal microbiota transplantation (FMT) mice. (b) Accumulation plot shows the relative abundance of phyla. (b) The firmicutes-to-bacteroidetes ratio was analysed for differences. (d) Co-occurrence network analysis depicts the relationship among taxonomic communities at the phylum level. (e) Important microbial taxa were identified using linear discriminant analysis effect size. (f) The relative abundance of differential bacteria was compared between the two groups. (g) Heatmap displays the association between key bacteria and clinical phenotypes. (h) Scatter plot shows the relationship between *Clostridium perfringens* and some phenotypes, including blood beta-hydroxybutyrate (BHB), body weight loss (BWL), and liver triacylglycerol (TAG). **p < 0.01, *p < 0.05.

To assess the availability of FMT, we examined the differences in gut microbiota diversity between recipient and FMT mice. The results indicated a significant difference in the Chao 1 and Shannon indices between recipient and FMT mice on a KD (Supplementary Figure S6). Unlike the recipient mice, the gut microbial community structure in donor mice was significantly altered following FMT (Figure 5a). Moreover, there was a significant difference in gut microbial community diversity between the FMT-HE and FMT-HYK groups, with both the Chao 1 and Shannon indices increasing in the FMT-HYK group. At the phylum level, core bacterial compositions were similar between the two groups (Figure 5b, c). However, the co-occurrence network analysis showed distinct clusters of microbial interactions in the FMT-HE and FMT-HYK groups (Figure 5d). The FMT-HYK group consisted of 415 nodes and 3,984 edges (average degree 19.2), which exceeded that of the FMT-HE group (169 nodes and 580 edges; average degree 6.86; Supplementary Table S6). The individual degree distribution of the nodes was significantly higher in the FMT-HYK group than in the FMT-HE group. In contrast, the FMT-HYK group exhibited a lower clustering coefficient (0.76) and a higher average path length (6.67), indicating a less active community with weaker microbial interactions. The LEfSe plot showed that *Muribaculaceae* and *Muribaculum* were the biomarkers in the FMT-HE group (Figure 5e). In the FMT-HYK group, significant biomarkers included *Clostridium sensu stricto 1*, *Escherichia Shigella*, *Odoribacter*, *Erysipelatoclostridium*, and *Dubosiella*. The relative abundance of *Clostridium perfringens*, *Clostridium cocleatum*, and *Escherichia coli* was significantly higher in the FMT-HYK group than in the FMT-HE group (Figure 5f). *C. perfringens* showed strong positive correlations with the levels of BHB (*r* = 0.66, *p* = 0.005) and liver TAG (*r* = 0.69, *p* = 0.003; Figure 5g, h).

### Commensal C. perfringens enhances ketogenesis and TAG accumulation during hyperketonemia

**Figure 6.**
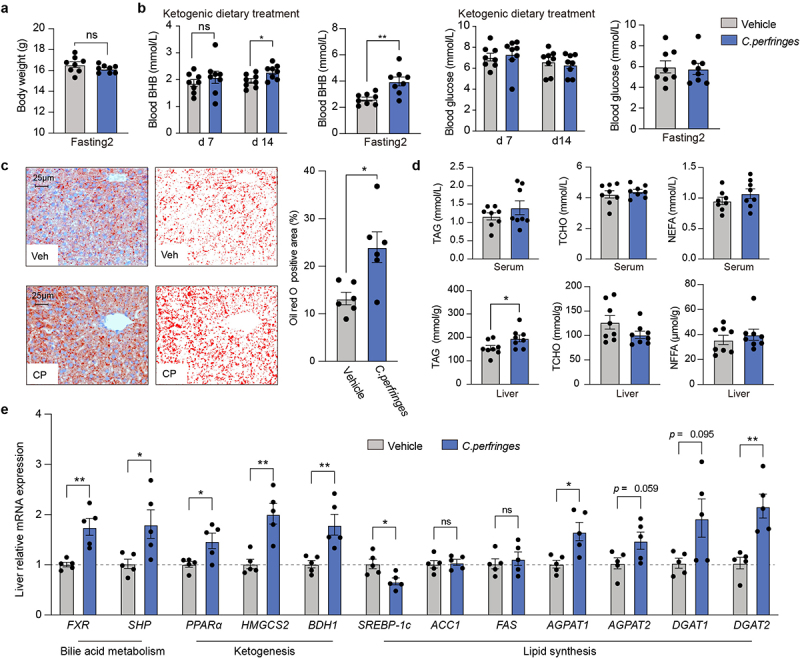
*Clostridium perfringens* aggravates the hepatic ketogenesis and lipid accumulation. (a) Body weight in each group were monitored during subsequent fasting following KD treatment. (b) The levels of blood beta-hydroxybutyrate (BHB) and glucose were measured on days 7 and 14 during ketogenic dietary treatment and fasting. (c) Oil red O staining (400×) was performed to assess lipid accumulation (n = 6 per condition). (d) Serum and liver levels of triacylglycerol (TAG), total cholesterol (TCHO), and non-esterified fatty acids (NEFA) were measured (n = 8 per condition). (e) Relative mRNA expression of key hepatic genes involved in ketogenesis and lipid synthesis were analysed (n = 5 per condition). Data represents mean ± SEM. **p < 0.01, *p < 0.05, ^ns^*p* > 0.05.

A retrospective analysis comparing hepatic ketogenesis between the PKD and AKD groups showed a strong correlation among *C. perfringens*, circulating BHB, and SBA (Supplementary Figure S7). Our findings revealed that *C. perfringens*, a gut commensal bacterium, belongs to toxinotype A, as determined based on isolation and identification techniques (Supplementary Figure S8). To further explore the regulatory effects of *C. perfringens* on hepatic ketogenesis and TAG accumulation, an intervention trial was conducted in the mice fed a KD (Supplementary Figure S9). No significant differences in body weight were observed between the vehicle and *C. perfringens* groups during the KD intervention (Figure 6a). Although glucose level remained unchanged, blood BHB level were notably elevated in the *C. perfringens* group after consumption and fasting (Figure 6b). Furthermore, *C. perfringens* intervention aggravated TAG accumulation in the liver (Figure 6c,d). Consistent with the FMT treatment results, the expressions of *FXR* and *SHP* mRNA were significantly upregulated in the *C. perfringens* group compared to that in the vehicle group (Figure 6e). In the ketogenesis pathway, key genes, including *PPARα*, *HMGCS2*, and *BDH1*, were significantly upregulated following *C. perfringens* intervention. The relative expression of *SREBP-1c* mRNA was decreased in the *C. perfringens* group; however, no differences were found in its downstream target genes, *ACC1* and *FAS*, between the two groups. In the glycerol phosphate-mediated TAG synthesis pathway, the *AGPAT1*, *AGPAT2*, *DGAT1*, and *DGAT2* mRNA levels were up-regulated in the *C. perfringens* group.

### Commensal Clostridium perfringens disturbs secondary bile acid metabolism

**Figure 7.**
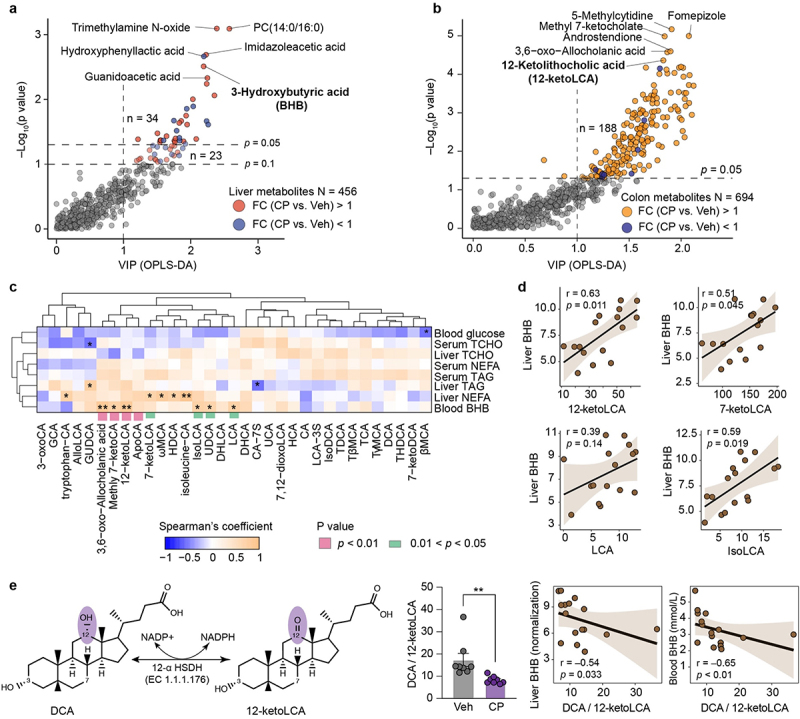
*Clostridium perfringens* contributes to secondary bile acids metabolism. (a) and (b) Scatter plots illustrate the differential metabolites in liver and colonic contents, respectively, based on *p* values and VIP from OPLS-DA. (c) The heatmap displays the correlation analysis between metabolic phenotypes and bile acid profiles in colonic contents, evaluated using Spearman’s coefficient. (d) Association between level of 3-hydroxybutyric acid (BHB) in liver and levels of lithocholic acid (LCA) species in colonic contents. Data on BHB in liver is presented as a normalised metabolomics feature. (e) Schematic diagram illustrating the metabolism of deoxycholic acid (DCA) to 12-ketoLCA. The ratio of DCA to 12-ketoLCA between the vehicle (Veh) and *C. perfringens* (CP) groups was analysed using the Mann – Whitney test. Correlation analysis between hepatic or blood BHB levels and DCA to 12-ketoLCA ratio was performed.

The results of PCA and OPLS-DA revealed a distinct separation in metabolomics profiles between the vehicle and *C. perfringens* groups (Supplementary Figure S10a and Figure S11a). In total, 456 and 694 metabolites were identified in the liver and colonic content, respectively (Supplementary Table S7 and Table S8). In the liver, levels of glycolithocholic acid 3-sulfate (5.82-fold), TCDCA (5.19-fold), GCA (2.88-fold), and TCA (1.99-fold) were higher in the *C. perfringens* group than that in the vehicle group (Supplementary Figure S10b). A total of 34 differential metabolites were identified between the two groups based on a VIP greater than 1 and a significance level of *p* < 0.05, primarily associated with pathways related to amino acid metabolism (Figure 7a and Supplementary Figure S10c). The liver levels of PC (14:0/16:0), trimethylamine N-oxide, and BHB were markedly higher in the *C. perfringens* group than in the vehicle group, exhibiting a strong correlation with serum BHB level (Supplementary Figure S10d). Concerning bile acid metabolism, the levels of primary bile acids (CA, GCA, TCA, CDCA, TCDCA, and TβMCA) did not significantly differ between the two groups (Supplementary Figure S10e). In the colon, the *C. perfringens* group exhibited increased levels of oxooctanoylcarnitine (17.38-fold), histamine (12.29-fold), and phenylacetylglycine (11.66-fold) compared to those in the vehicle group (Supplementary Figure S11b). A total of 188 differential metabolites were identified in the colon between the two groups, which were involved in sphingolipid metabolism, histidine metabolism, caffeine metabolism, and arginine biosynthesis, and reflected a co-metabolic interaction between the host and microbiota (Figure 7b, Supplementary Figure S11c). Specifically, the level of 12-ketoLCA was significantly elevated in the *C. perfringens* group compared to the vehicle group, along with increased levels of 7-ketoLCA, isoLCA, and UDCA in the *C. perfringens* group. The levels of 12-ketoLCA and isoLCA in the colonic content were positively correlated with both circulating and hepatic BHB levels (Figure 7c,d). Although 7-ketoLCA was found in both the liver and colon, the colonic 7-ketoLCA showed a strong positive correlation with hepatic BHB levels. Of note, the DCA to 12-ketoLCA ratio was significantly lower in the *C. perfringens* group, indicating an increased oxidative capacity of bile acids in the gut microbiota following colonization with *C. perfringens* (Figure 7e). This decrease in the DCA to 12-ketoLCA ratio was strongly negatively correlated with elevated levels of hepatic and blood BHB.

## Discussion

The potential regulatory mechanism of the gut microbiota on hepatic ketogenesis and steatosis during KD-induced hyperketonemia, incorporating various interventions, was explored in this study. We observed that the KD mitigated body weight loss following long-term fasting but elevated the circulating BHB levels and liver lipid accumulation. Additionally, consumption of the KD altered the composition and interactions within the gut microbial community, preventing gut microbial remodeling during Abx-induced depletion. The severity of hepatic ketogenesis and lipid accumulation correlated with alterations in bile acids metabolism through the liver-gut axis. Moreover, we identified *C. perfringens* as a key regulatory commensal bacterium that regulates hepatic ketogenesis during hyperketonemia by disturbing bile acid metabolism. Our findings elucidate the relationship between the gut microbiota and hepatic lipid metabolism during a KD consumption, offering novel insights into the metabolic regulatory mechanism of *C. perfringens* under altered metabolic environments with carbohydrate source conditions.

A KD differs from a traditional high-fat diet in its composition, being characterized by more lower carbohydrate and protein levels, and higher fat; however, hepatic lipid metabolism challenges occur in both high-fat and ketogenic diets, with the lipid accumulation and insulin resistance being more severe in the short-term consumption of a KD.^16,19^ Despite the presence of glucose, amino acids derived from dietary protein serve as a carbon source for fatty acid synthesis in the liver, particularly in *de novo* lipogenesis. A high-protein diet has been regarded as a substantial contributor to the development of metabolic disorders.^50,51^ Key genes involved in lipogenesis, such as *FAS*, *ACC1*, and *SCD1*, were downregulated in a very low-protein diet compared with those in a diet with an equivalent fat content that included 20% protein.^51^ However, a low-protein intervention trial demonstrated that the levels of hepatic TAG were significantly higher in a diet containing 5% protein compared to a diet with 15% protein.^52^ Although the dietary protein level is higher in the high-fat diet, a significant increase in liver lipid accumulation and steatosis also observed in mice fed a KD. Additionally, the study population indicated the KD exacerbated the hepatic lipid accumulation, particularly in individuals with normal weight who consumed the diet.^53^ Some intervention trials revealed that the decrease in very-low-density lipoprotein (VLDL) and inhibition of the VLDL receptor were important factors contributing to KD-induced hepatic lipid accumulation.^54^ Previous studies suggest that FXR activation can regulate VLDL secretion in the liver.^55^ In the present study, the expression of *DGAT2* and *FXR* mRNA were elevated in association with severe circulating BHB level and hepatic lipid accumulation. DGAT2, a member of the DGAT enzyme family, facilitates the conversion of fatty acid from *de novo* lipid synthesis into TAG, while DGAT1 aids in metabolizing exogenous fatty acids into TAG.^56^ Jornayvaz et al.^57^ discovered a marked increase in liver TAG levels and DGAT expression in mice on a KD. Previous research has found the overexpression of DGAT2 in hepatic steatosis, although inhibiting SREBP-1c cleavage can ameliorate hepatic lipid accumulation by inhibiting DGAT2 expression.^58^ While SREBP-1c regulates DGAT2 expression, a previous study found no significant change in liver SREBP-1c levels after 9 weeks on a KD compared to that of a 66 % calorie-restricted diet, suggesting that hepatic steatosis induced by a KD may not be modulated by SREBP-1c.^59^ We also found that the expression level of liver *SREBP-1c* mRNA did not significantly increase with the consumption of a KD across various interventions. Some studies have suggested that the KD may induce insulin resistance and hepatic lipid accumulation by activating the PKCε and IL-INK signaling pathways.^16,57^ In addition, FXR regulates *de novo* lipid synthesis by inhibiting the expression of *SREBP-1c*. ^60^ We observed that the *SREBP-1c* levels did not correlate with hepatic TAG accumulation in the ketotic state; however, the expression levels of *FXR*, *SHP*, *AGPAT2*, and *DGAT2* were markedly increased. Recent reports indicate that *SREBP-1c* expression does not influence the regulation of *FXR* and *SHP* during a high-fat diet, suggesting that altered levels of hepatic fatty acids are related to the FXR-dependent regulation of DAGT2 expression.^61,62^

Different pattern of diet has been confirmed to modify metabolic homeostasis by altering the composition of the gut microbial community.^21^ The high-fat diet may substantially impact the host metabolic health by shifting the gut bacterial ecosystem.^63,64^ Despite both the high-fat diet and KD containing a high level of fat, their impacts on gut microbiota differed. The high-fat diet, which consists of 75% fat, did not elevate the circulating level of BHB; but, the relative abundance of *Lactobacillus* and *Parasutterella* were altered in the gut compared to those with the KD, which consists of 90% fat.^26^ Additionally, intervention studies examining various dietary fat levels indicate a significant difference in the diversity of the gut microbial community between high-fat and ketogenic diets, and the relative abundance of *Actinobacteria* progressively increased with higher dietary fat levels; however, the relative abundances of *Bifidobacterium* and *Lactobacillus* were found to be lowest with the KD.^25,65^ Conversely, the gut microbiota also influences metabolic health during hyperketonemia.^17,22,23,65^ A recent study also shows that a KD alters the abundance of Firmicutes and Bacteroidetes in the intestines, while the BHB produced during host ketogenesis selectively suppresses the growth of *Bifidobacterium*.^25^ Kong et al.^24^ report an increase in *Akkermansia muciniphila*, a member of Verrucomicrobia, in the FMT of KD-fed mice. The KD protects intestinal barrier function and mitigates the inflammatory response in mice with ulcerative colitis. In our study, we also observed a significant increase in the relative abundance of *Akkermansia muciniphila* in the PKD group. The abundance of *Akkermansia muciniphila* was higher in the Abx pre-treatment groups than in the PKD group, indicating that *Akkermansia* became the dominant bacterium during the remodeling process of Abx-induced gut microbiota depletion, consistent with the findings of de Nies et al.^36^ Of note, we observed that the degree of hepatic lipid accumulation was higher in the PKD group than in the AKD group. An intervention trial suggests that the colonization of *Akkermansia muciniphila* increases L-aspartate levels, which ameliorates lipid accumulation and oxidation stress in the metabolic dysfunction-associated fatty liver by activating the LKB1-AMPK axis.^66^ Additionally, *Akkermansia muciniphila* alleviates hepatic steatosis and inflammation in mice on a high-fat diet by regulating the gut FXR-FGF15 signaling axis.^67^ However, whether *Akkermansia muciniphila* is a key bacterium in regulating hepatic lipid synthesis under a KD requires further investigation.

Bile acids serve as important metabolic messengers that regulate lipid metabolism homeostasis through the gut-liver axis.^33^ Bile acids are synthesized from cholesterol in the liver via both classical and alternative pathways, with the classical pathway predominantly producing 12-OH bile acids by activating CYP7A1 and CYP8B1 enzymes.^68^ In contrast, the alternative pathway produces non-12-OH bile acids through the action of CYP27A1 and CYP7B1. Additionally, glycine- or taurine-conjugated bile acids are synthesized from primary bile acids through the actions of bile acid-CoA synthase and bile acid-amino acid transferase activity in the liver. Upon entering the intestine, these conjugated bile acids are deconjugated by gut microbiota.^69^ In the bile acid synthetic pathway, a significant alteration in the ratios of 12-OH to non-12-OH bile acids and conjugated to unconjugated bile acids are considered as biomarkers of metabolic diseases.^70^ Bile acids metabolism disorders are closely related to high-fat-induced hepatic lipid accumulation, accompanied by a significant up-regulation of *CYP8B1* mRNA in the liver,^61,71^ consistent with our findings. FXR is the primary receptor for bile acids, and its activation in the liver promotes SHP expression to inhibit CYP7A1 and CYP8B1, maintaining the synthetic homeostasis of hepatic bile acids.^70,72^ Furthermore, non-12-OH bile acids in the intestine can activate FXR and induce the secretion of FGF15/19, thereby inhibiting the expression of *CYP8B1* and *CYP7B1* in the liver.^73,74^ Our study revealed no significant effects of the KD on hepatic FXR and SHP, consistent with the findings of Hori et al.^61^ We also observed increased levels of serum primary bile acids, including TCA, TCDCA, and TβMCA, with KD consumption. Additionally, the ratio of non-conjugated to conjugated bile acids markedly decreased in the AKD group, indicating a reduced capacity for secondary bile acid metabolism during a KD consumption due to manipulated gut microbiota. The ability of hepatic ketogenesis in response to the KD shows differences in the composition of the gut microbial community, correlating with microbial bile acid and hepatic *FXR* expression levels. In the hepatic ketogenesis pathway, HMGCS2 and BDH1 serve as key rate-limiting enzymes by the coordinated actions of PPARα and FXR.^75,76^ We also found that *FXR* and *PPARα* mRNA expression levels were higher in the *BDH1* overexpressing group during prolonged ketosis. Additionally, *PPARα* regulates hepatic FGF21 levels in ketotic states, while FGF21 overexpression observably enhances the BDH1 level during a high-fat diet consumption.^77,78^ Consequently, the BA-FXR-PPARα signaling pathway emerges as a potential regulatory mechanism for ketogenic capacity in hyperketonemia conditions.

*Clostridium*, as important commensal bacteria, participates in the regulation of metabolic health by disrupting bile acids metabolism.^79,80^ Previous studies have reported that oral intervention with *C. sporogenes* promotes hepatic lipid accumulation by up-regulating the expression of genes involved in lipid synthesis and enhancing energy absorption.^29^ A recent study by She et al.^40^ shows that the colonization of *Clostridium* in the intestine facilitates the conversion of CDCA into UDCA and 7-ketoLCA. This process stimulates intestinal L cells to secrete GLP-1 for glucose homeostasis and ameliorates metabolic disorders. Here, we identified *C. perfringens* type A as a commensal bacterium associated with KD-induced hyperketonemia, with its relative abundance strongly correlating with levels of blood BHB and liver TAG. *C. perfringens* is a gram-positive anaerobic bacterium typically present in the intestines, where it can cause clinical diarrhea and bloating when it proliferates excessively.^39,81^ While previous studies have concentrated on the pathogenesis of *C. perfringens*, its role in metabolic disorders remains less explored. Conversely, numerous studies report that the colonization of *C. difficile*, *Clostridium. sp*, and *C. paraputrificum* regulates metabolic health by producing short-chain fatty acids and bile acids.^40,82,83^ Guzior et al.^80^ showed that conjugated primary bile acid is metabolized by the bile salt hydrolase/transferase of *C. perfringens* at an acidic pH, indicating the ability of the bacterium to produce bile acids microbiologically. Metabolomics analysis revealed significantly increased levels of secondary bile acids in the colonic contents of the mice orally administrated with *C. perfringens*. Particularly, increases in 7-ketoLCA, 12-ketoLCA, and isoLCA levels strongly positively correlated with circulating BHB level. Additionally, colonization by *C. perfringens* enhances hepatic ketogenesis during hyperketonemia by upregulating the expression of *FXR*, *SHP*, *PPARα*, *HMGCS* and *BDH1* mRNA. A clinical cohort study reports that the level of faecal 12-ketoLCA is significantly higher in patients with type 2 diabetes.^84^ In the keto-BA metabolism pathway, CDCA is converted to 7-ketoLCA by the action of 7alpha- hydroxysteroid dehydrogenase (7α-HSDH), while 12-ketoLCA is metabolized from DCA under the action of 12α-HSDH.^83,85^ Early studies by Macdonald et al.^86^ have described the activities of 3α-, 7α- and 12α-HSDH in *C. perfringens*. Regarding potential mechanisms, a previous study shows that 7-ketoLCA, a gut FXR antagonist, attenuates aspirin-induced intestinal damage by facilitating Wnt signaling. For 12-ketoLCA, it suppresses the secretion of IL-17A in mice with ulcerative colitis by activating the VDR.^87^ Alimov et al.^88^ also reported the weak inflammasome activity of 12-ketoLCA. In addition, the constitutive androstane receptor is targeted by the 12-ketoLCA nuclear receptor, regulating hepatic lipid oxidation through overlaps with PPARα.^89^ However, it remains to be determined whether the ketoLCA-FXR axis is a key regulatory pathway through which *C. perfringens* influences hepatic ketogenesis during hyperketonemia, necessitating further investigation in patient-derived organoid models. Besides hepatic ketogenesis, we found that colonization by *C. perfringens* exacerbates TAG accumulation by enhancing the expression levels of *AGPAT1*, *AGPAT2*, and *DGAT2* mRNA. AGPAT, particularly AGPAT1 and AGPAT2, are the major subtypes and key regulatory enzymes in TAG synthesis within the glycerol-3-phosphate pathway, controlling the conversion of lysophosphatide to phosphatid.^90^ Bradley et al.^91^ revealed a significant increase in the liver AGPAT2 expression of mice after fasting for 16 h. This increase accompanies elevated blood levels of BHB, suggesting a potential correlation between ketogenesis and lipid synthesis mediated by AGPAT with varying ketogenic capacities. Thus, *C. perfringens* is a key commensal bacterium involved in the regulation of hepatic ketogenesis and lipid synthesis during KD-induced alterations in the gut microbial ecosystem. Future use of germ-free animal models will help further explore the specific effects of *C. perfringens* on hepatic lipid metabolism.

**Figure 8.**
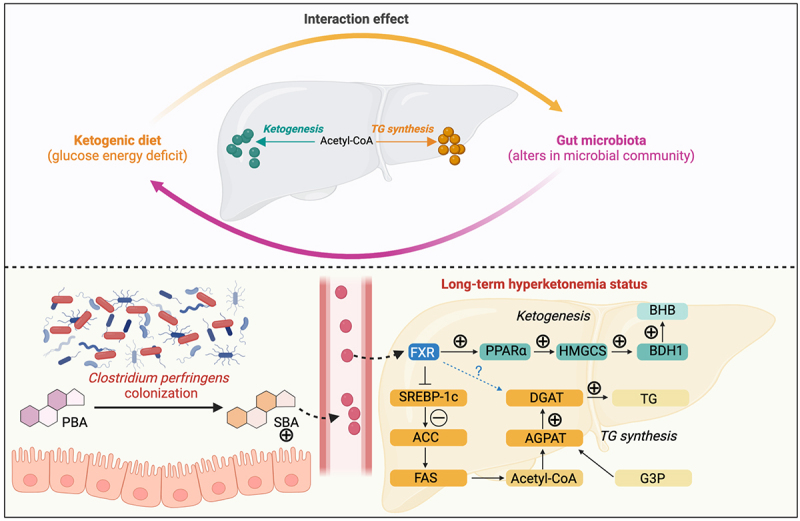
Integrative diagram illustrating the regulatory mechanisms by which the gut microbiota influences hepatic ketogenesis and *de novo* lipid synthesis by disrupting bile acid metabolism during ketogenic diet induced-hyperketonemia (Created with BioRender.com). PBA, primary bile acids. SBA, secondary bile acids. TG, triacylglycerol. BHB, β-hydroxybutyrate. G3P, glycerol-3-phosphate.

In summary (Figure 8), the capacities of hepatic ketogenesis and lipid synthesis are regulated by the interaction between a KD and gut microbiota. Alterations in the gut microbial community facilitate the colonization of *C. perfringens*, a commensal bacterium linked to increased hepatic levels of BHB and TAG, during KD-induced hyperketonemia. Our findings suggest that altered secondary bile acid metabolism, particularly the conversion of DCA into 12-ketoLCA, significantly influences hepatic metabolic homeostasis in the ketotic states.

## Supplementary Material

Supplemental Material

## Data Availability

The 16S rRNA sequences of cattle fecal samples have been deposited into the NCBI Sequence Read Archive (SRA) under the accession numbers PRJNA1173372 (https://dataview.ncbi.nlm.nih.gov/object/PRJNA1173372?reviewer=jro1be46mip631f6q9fn4sjj4e.) and PRJNA1173382 (https://dataview.ncbi.nlm.nih.gov/object/PRJNA1173382?reviewer=3cljg2nr4ch3hrhjggf95136e6).
