# Supplementary material for: Gut microbiota regulates hepatic ketogenesis and lipid accumulation in ketogenic diet-induced hyperketonemia by disrupting bile acid metabolism
Source: Gut Microbes. 2025 Apr 23;17(1):2496437. doi: 10.1080/19490976.2025.2496437 (PMC12026136; doi:10.1080/19490976.2025.2496437)
Supplement: Supplemental Material [file KGMI_A_2496437_SM8307.zip › Supplementary materials.docx]

*Supplementary materials*

**Gut microbiota regulates hepatic ketogenesis and lipid accumulation in ketogenic diet-induced hyperketonemia by disrupting bile acid metabolism**

Zhengzhong Luo,^a,b^ Yixin Huang,^a,b^ Kang Yong,^c^ Dan Wu,^a,b^ Linfeng Zheng,^a,b^ Xueping Yao,^a,b^ Liuhong Shen,^a,b^ Shumin Yu,^a,b^ Baoning Wang ^d*^ and Suizhong Cao ^a,b*^

^a^College of Veterinary Medicine, Sichuan Agricultural University, Chengdu 611130, China

^b^Key Laboratory of Animal Disease and Human Health of Sichuan Province, Chengdu 611130, China

^c^College of Animal Science and Technology, Chongqing Three Gorges Vocational College, Chongqing 404100, China

^d^West China School of Basic Medical Sciences and Forensic Medicine, Sichuan University, Chengdu 610041, China

*Correspondence author:

Baoning Wang, wangbn@scu.edu.cn

Suizhong Cao, suizhongcao@sicau.edu.cn

Zhengzhong Luo, Yixin Huang, and Kang Yong contributed equally to this article.

**Supplementary Figure**


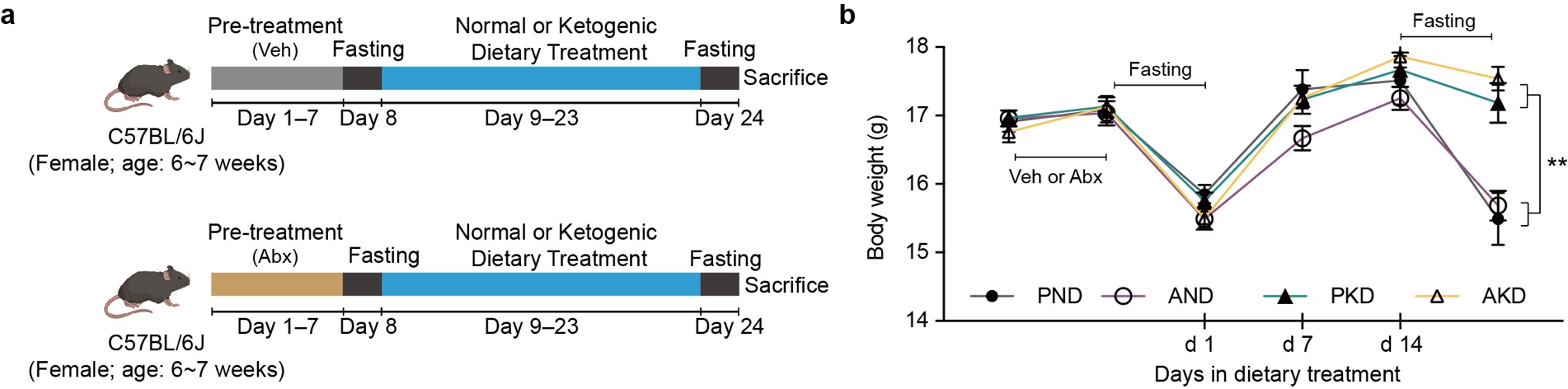


**Figure S1.** (a) Schematic representation of normal and ketogenic dietary treatments following different interventions. (b) Changes in body weight were monitored across all groups throughout the experimental period. Veh, vehicle. Abx, antibiotics. ^**^*p* < 0.01.


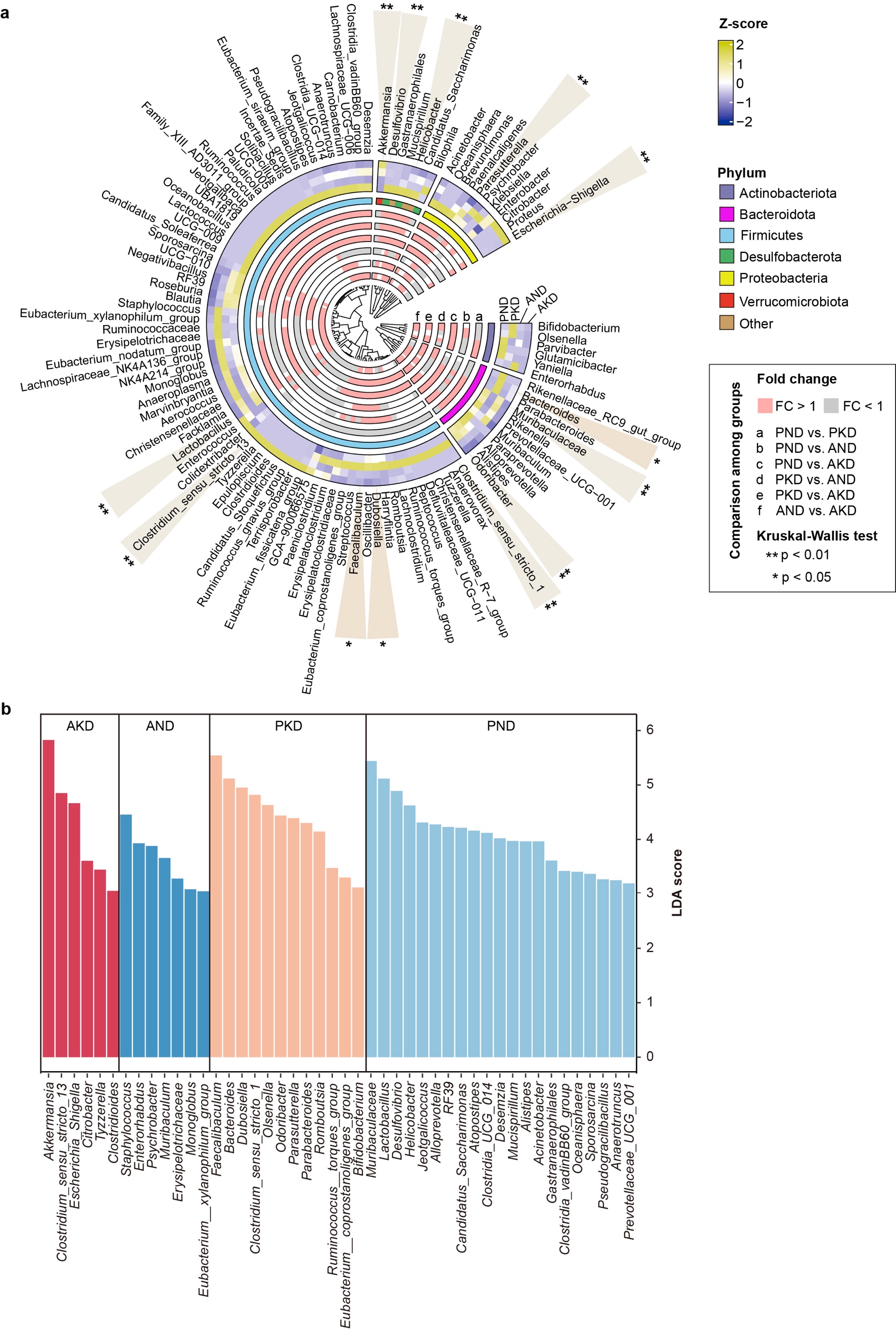


**Figure S2.** Changes in the relative abundance of genus taxa in response to ketogenic diet and Abx treatment. (a) The circular heatmap illustrates the alterations in the relative abundance of genus taxa across different treatments. Differential genus analysis is performed using the Kruskal–Wallis test with the Benjamini–Hochberg adjustment, focusing on genera with a mean relative abundance greater than 1%. (b) The histogram displays the analysis scores of linear discriminant analysis effect size (LEfSe) in genera taxa (only LEfSe scores > 3 are shown). Statistical significance was denoted as ^**^*p* < 0.01, ^*^*p* < 0.05.


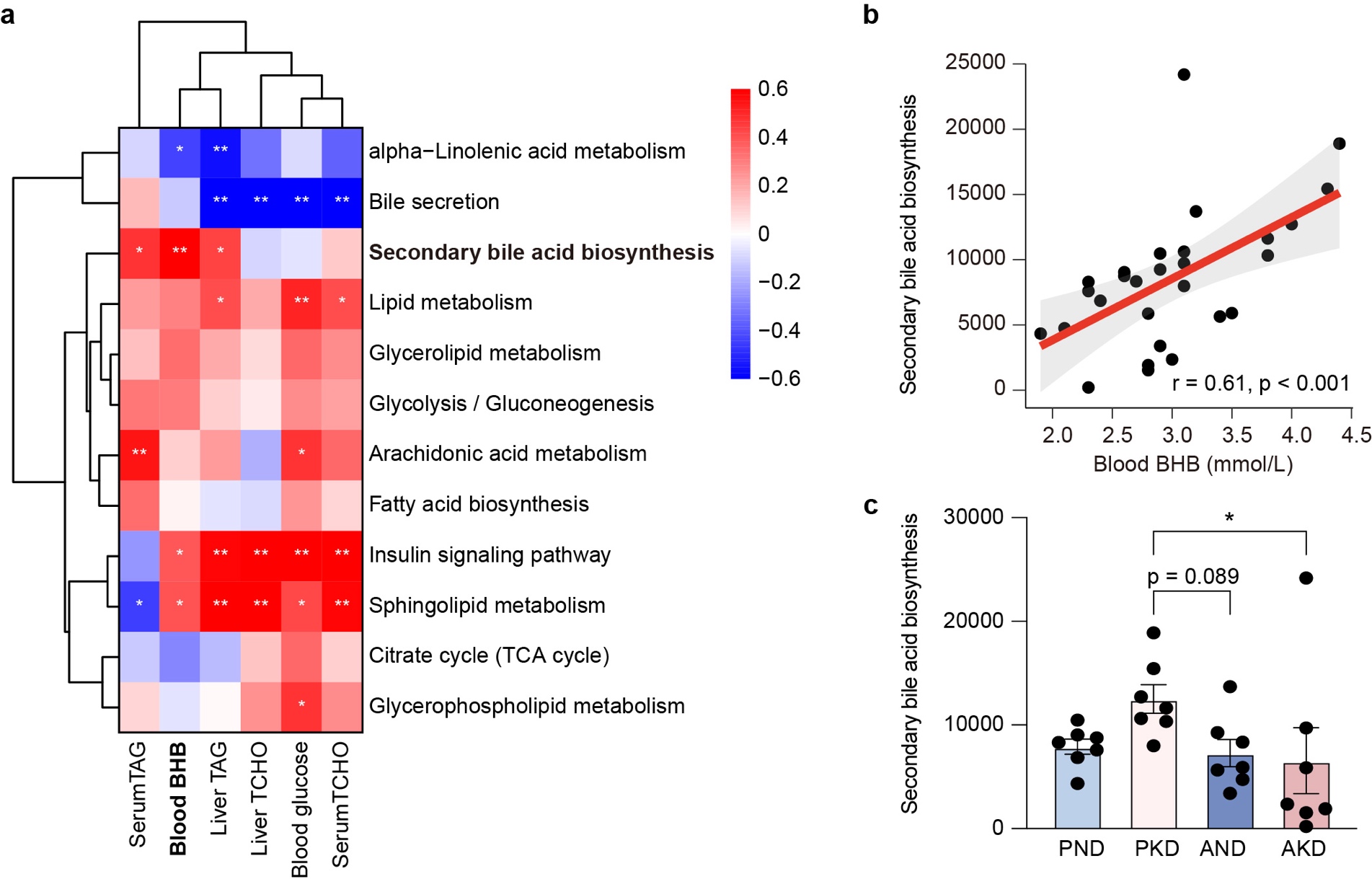


**Figure S3**. Gut microbial function related with the altered in clinical phenotypes. (a) Heatmap displays the association between the glucolipid metabolism-related pathway in KEGG level 3 and phenotypes of individuals after dietary intervention based on Spearman’s correlation. (b) The relationship between the abundance of secondary bile acid biosynthesis and the level of blood beta-hydroxybutyrate (BHB). (c) Differences among the four groups were assessed using the Kruskal-Wallis test with Benjamini-Hochberg adjustment. Statistical significance was denoted as ^**^*p* < 0.01, ^*^*p* < 0.05.


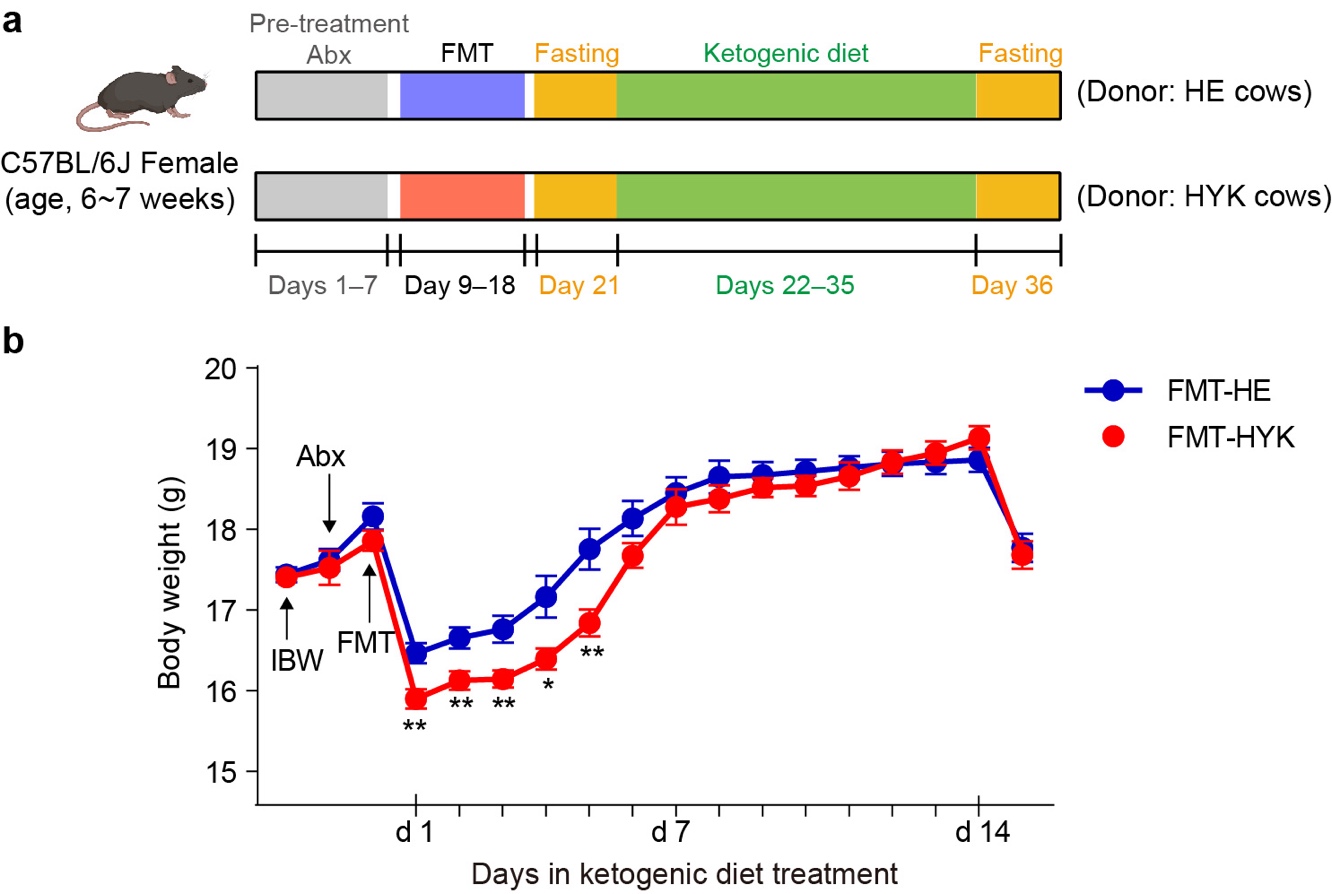


**Figure S4**. Faecal microbiota transplantation (FMT) from various dairy cow donors. (a) Overview of the FMT process and ketogenic dietary intervention, with faecal donors sourced from healthy (HE) and hyperketonemic (HYK) dairy cows. (b) Comparison of body weight changes between the FMT-HE and FMT-HYK groups at various time points. IBW, initial body weight. Abx, antibiotics. Data represents mean ± SEM. ^**^p < 0.01, ^*^p < 0.05.


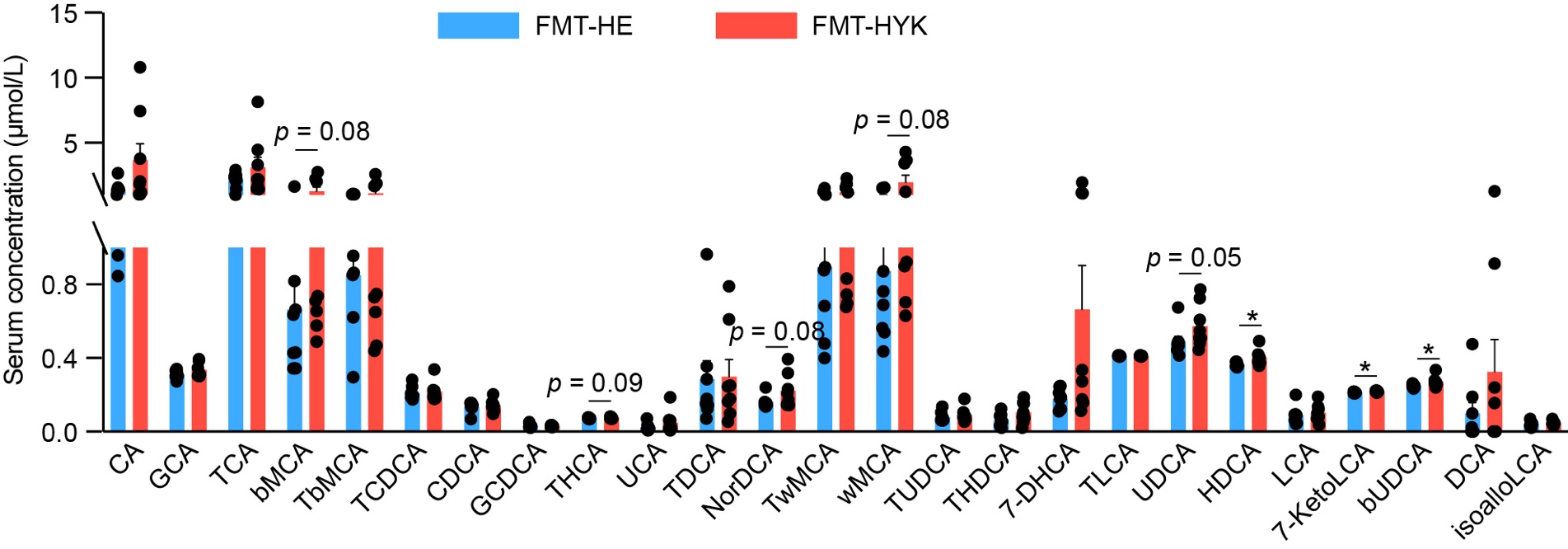


**Figure S5**. The level of individual bile acid in serum between the FMT-HE and FMT-HYK groups. ^*^*p* < 0.05.


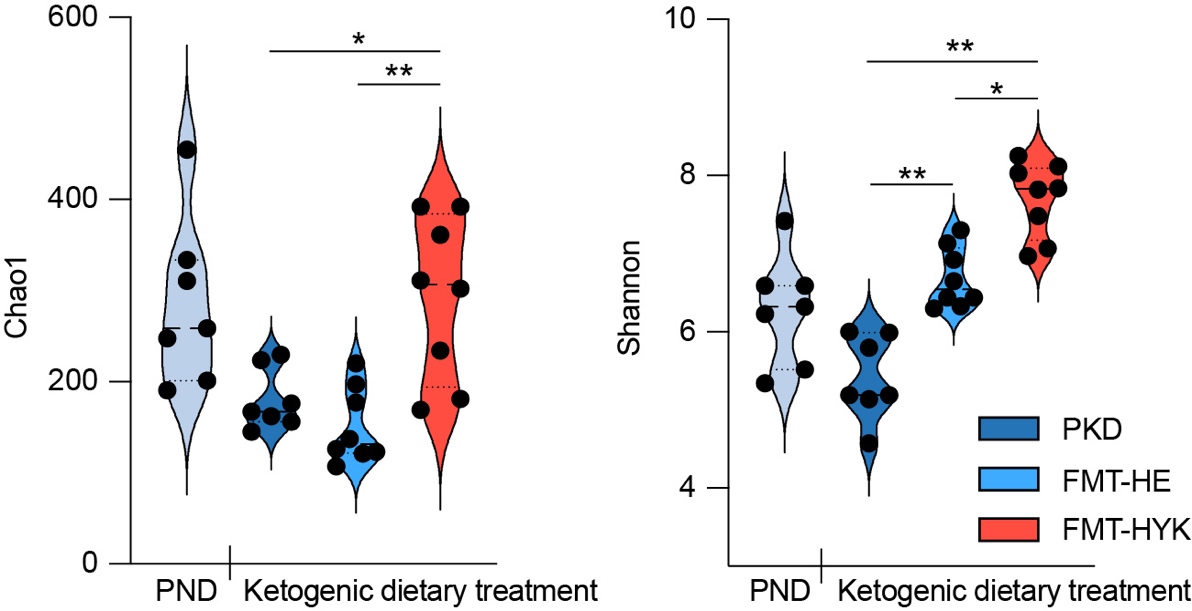


**Figure S6**. The alpha diversity of gut microbiota was compared between recipient and faecal microbiota transplantation (FMT) mice. A significant analysis was performed among three groups: the ketogenic diet (PKD), fecal microbiota transplantation with healthy donor (FMT-HE), and fecal microbiota transplantation with hyperketonemic donor (FMT-HYK), using the Kruskal-Wallis test.


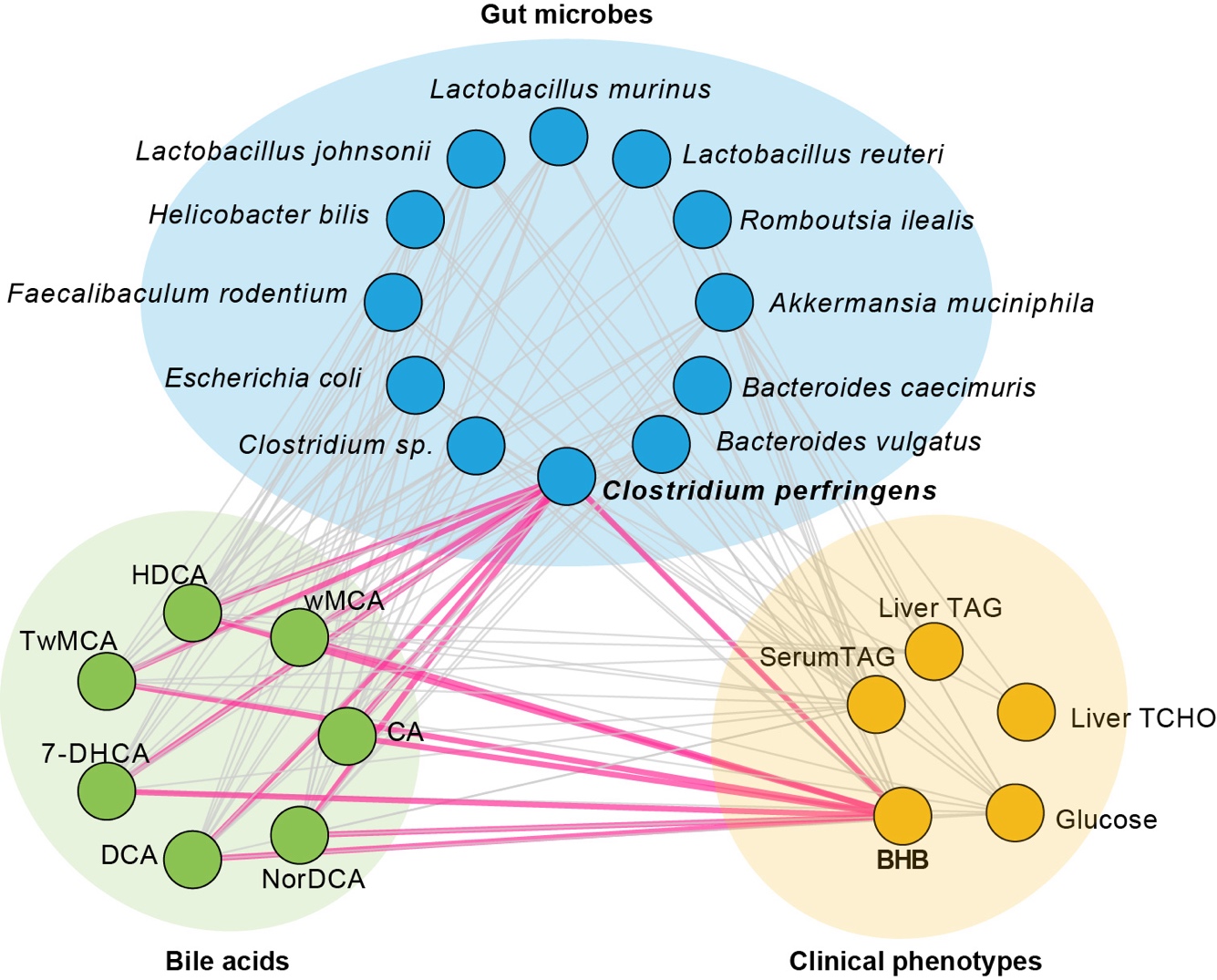


**Figure S7**. Correlation networks among the gut bacteria, bile acids and clinical phenotypes based on Spearman’s coefficient. Only correlations with r values greater than 0.5 or less than -0.5 and with a p value adjusted to below 0.05 are included in the analysis. The significant differences in gut microbes and bile acids between the PKD and AKD groups were incorporated into network analysis.

**
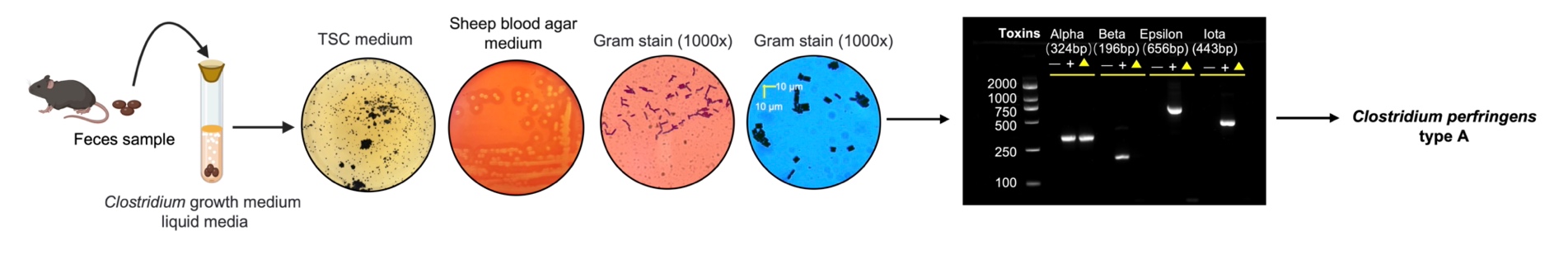
**

**Figure S8**. Isolation and identification of *Clostridium perfringens* in gut of mice. Symbol ‘-’ indicate the negative control, ‘+’ indicate the positive control, and ‘△’ represent the targeted sample. The positive control samples included various toxin strains of *Clostridium perfringens* preserved in our research group’s laboratory.


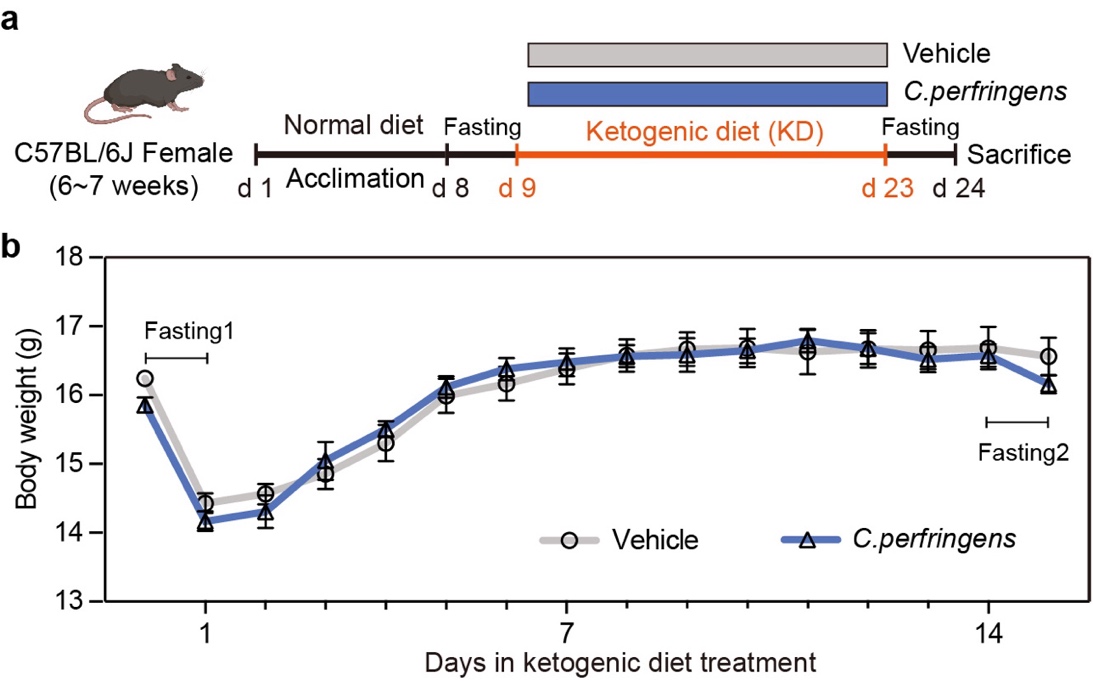


**Figure S9**. (a) Schematic representation of the *C. perfringens* and ketogenic dietary treatment intervention. (b) Body weight changes in each group were monitored throughout the experimental period.


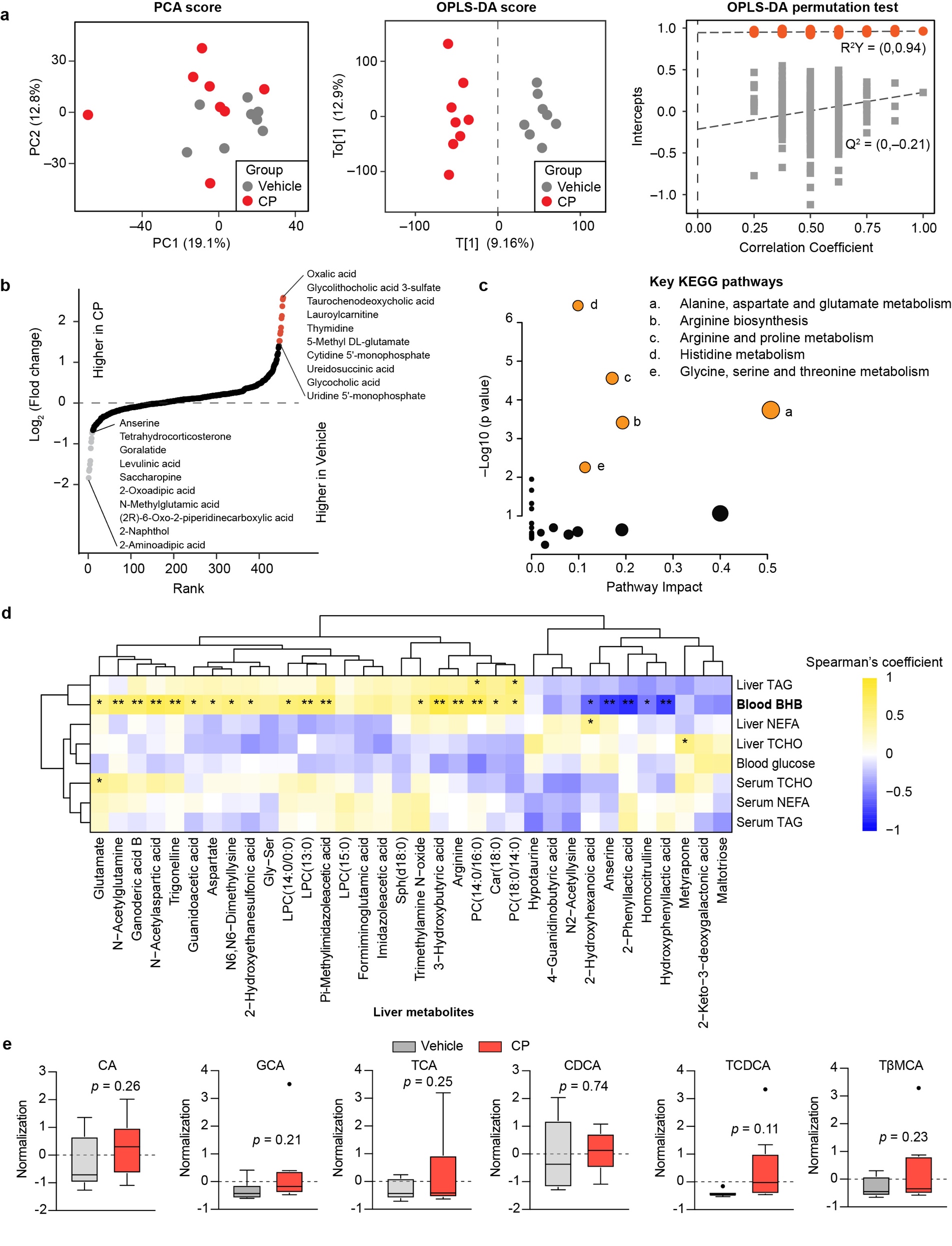


**Figure S10.** *Clostridium perfringens* alters the metabolic profiles in liver. (a) Multidimensional statistical analysis was performed, including principal component analysis (PCA), orthogonal partial least squares discriminant analysis (OPLS-DA), and permutation test of OPLS-DA. (b) Fold change (FC) analysis of metabolites in liver. FC greater than 0 indicates relatively higher concentration in the *C. perfringens* (CP) group, whereas FC less than 0 indicates a lower concentration in CP group than in vehicle group. (c) Topological analysis of differential metabolites in the KEGG pathway was conducted. (d) The heatmap show the association between differential metabolites in the liver and host metabolic phenotypes based on Spearman’s coefficient. (e) A comparison of primary bile acids in the liver between the vehicle and CP groups was carried out using Student’s t-test after data normalization.


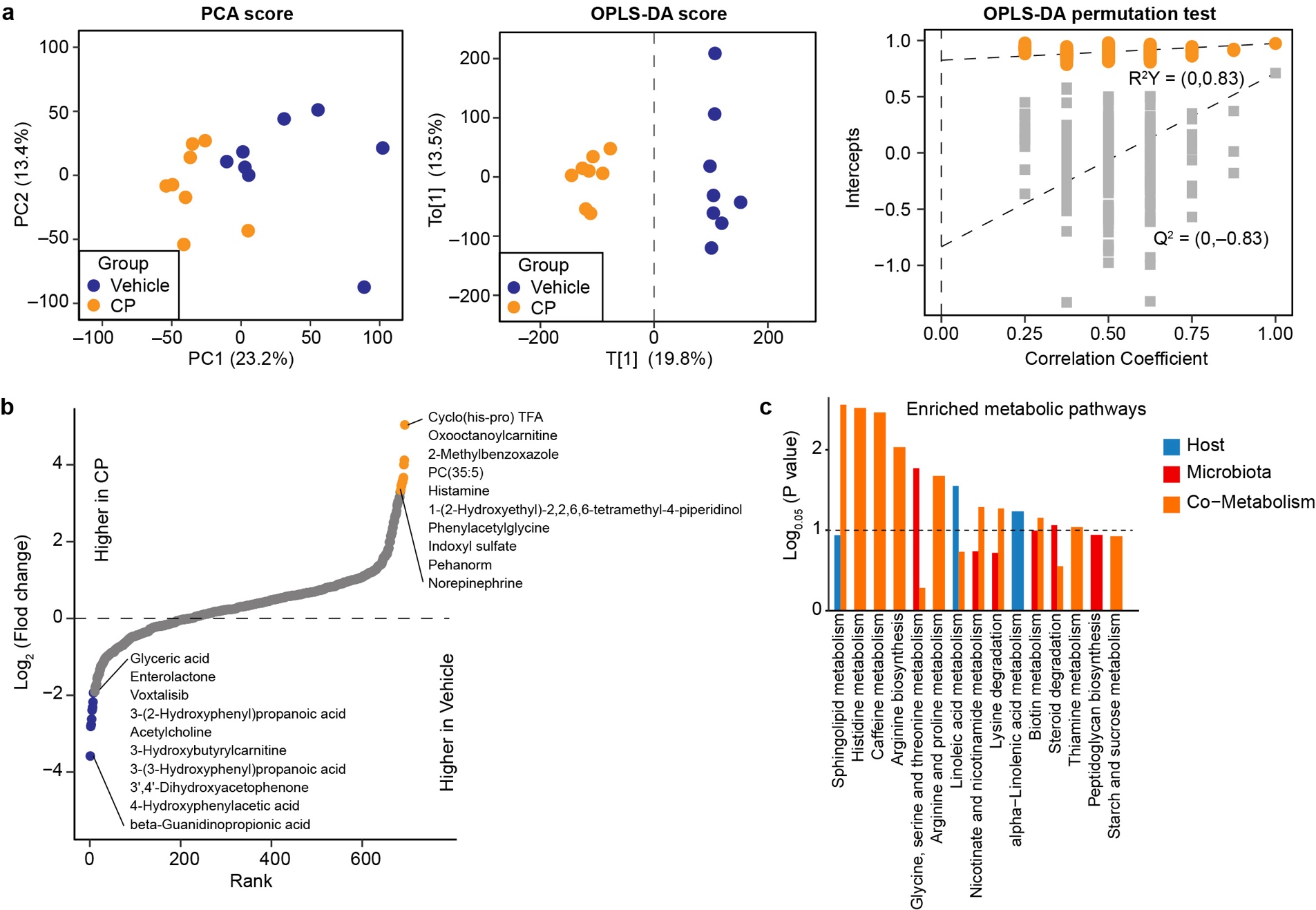


**Figure S11.** *Clostridium perfringens* alters the metabolic profiles in colonic contents. (a) Multidimensional statistical analysis was performed, including principal component analysis (PCA), orthogonal partial least squares discriminant analysis (OPLS-DA), and permutation test of OPLS-DA. (b) Fold change (FC) analysis of metabolites in colonic contents. FC greater than 0 indicates relatively higher concentration in the *C. perfringens* (CP) group, whereas FC less than 0 indicates a lower concentration in CP group than in vehicle group. (c) Enriched metabolic KEGG pathways of differential metabolites was conducted using MetOrigin 2.0 platform (https://metorigin.met-bioinformatics.cn/).

**Supplementary Table**

**Table S1.** The ingredient and nutritional levels of control and ketogenic diets

| Items | Control diet | | Ketogenic diet | |
| --- | --- | --- | --- | --- |
|  | gm, % | kcal, % | gm, % | kcal, % |
| Ingredient |  |  |  |  |
| Casein | 100 | 400 | 100 | 400 |
| L-cystine | 1.5 | 6 | 1.5 | 6 |
| Corn starch | 371 | 1484 | 0 | 0 |
| Maltodextrin | 35 | 140 | 0 | 0 |
| Sucrose | 406 | 1625 | 0 | 0 |
| Cellulose, BW 200 | 50 | 0 | 50 | 0 |
| Soyben oil | 25 | 225 | 25 | 225 |
| Cocoa butter | 20 | 180 | 381 | 3429 |
| Mineral mix, S10026 | 50 | 0 | 50 | 0 |
| Vitamin mix, V10001C | 1 | 4 | 1 | 4 |
| Choline bitartrate | 2 | 0 | 2 | 0 |
| FD＆C Yellow dye #5 | 0 | 0 | 0.025 | 0 |
| FD＆C Red dye #40 | 0.025 | 0 | 0.025 | 0 |
| FD＆C Blue dye #1 | 0.025 | 0 | 0 | 0 |
| Total | / | 4064 | / | 4064 |
| Nutritional level |  |  |  |  |
| Fat | 4.24 | 10.06 | 16.63 | 89.99 |
| Carbohydrate | 76.5 | 79.94 | 0 | 0.01 |
| Protein | 9.56 | 10 | 66.5 | 10 |
| Kcal/gm | 3.8 | / | 6.7 | / |

**Table S2**. The information of PCR primer sequence in *Clostridium perfringens*

| Primer name | Primer sequence  (5'-3') | Fragment size (bp) | Annealing temperature (℃) |
| --- | --- | --- | --- |
| 27F | AGAGTTTGATCCTGGCTCAG | 1465 | 55 |
| 1492R | GGTTACCTTGTTACGACTT |  |  |

**Table S3.** The information of primer in differential *Clostridium perfringens* toxins

| Gene name | | Primer sequence  (5'-3') | Fragment size  (bp) | Annealing temperature (℃) |
| --- | --- | --- | --- | --- |
| *Cpa* | F | GCTAATGTTACTGCCGTTGA | 324 | 53 |
|  | R | CCTCTGATACATCGTGTAAG |  |  |
| *Cpb* | F | GCGAATATGCTGAATCATCTA | 196 |  |
|  | R | GCAGGAACATTAGTATATCTTC |  |  |
| *Etx* | F | GCGGTGATATCCATCTATTC | 656 |  |
|  | R | CCACTTACTTGTCCTACTAAC |  |  |
| *Ia* | F | ACTACTCTCAGACAAGACAG | 443 |  |
|  | R | CTTTCCTTCTATTACTATACG |  |  |

**Table S4.** The information of qPCR primer sequence in mice

| Gene name | Forward sequence (5' to3') | Reverse sequence (5' to3') |
| --- | --- | --- |
| *FXR* | CCCCTGCTTGATGTGCTAC | CGTGGTGATGGTTGAATGTC |
| *SHP* | AAGGGCACGATCCTCTTCAA | CTGTTGCAGGTGTGCGATGT |
| *CYP7A1* | CTGGGCTGTGCTCTGAAGT | GGGAGTTTGTGATGAAGTGGA |
| *CYP8B1* | ACAGCGTGATGGAGGAGAGT | AGGGGAAGAGAGCCACCTTA |
| *CYP27A1* | TCCCAGTGTCTTTCCTGAGC | CACAGAGCCGAATGGATGTA |
| *CYP7B1* | TGAGGTTCTGAGGCTGTGC | TGGAGGAAAGAGGGCTACAA |
| *TGR5* | GCCCAAAGGTGTCTACGAGT | TCAAGTCCAGGTCAATGCTG |
| *PPARα* | AGAGCCCCATCTGTCCTCTC | ACTGGTAGTCTGCAAAACCAAA |
| *Hmgcs2* | GGTGTCCCGTCTAATGGAGA | ACACCCAGGATTCACAGAGG |
| *BDH1* | GAATTCAGCCTGCCGGTTTG | TGCATCCCGCTGTCAGGTAA |
| *ACAT1* | GTCTGGCTAGTATTTGCAACG | TTCAGCCGGTCACATGG |
| *SREBP-1c* | ATCGCAAACAAGCTGACCTG | AGATCCAGGTTTGAGGTGGG |
| *ACC1* | ATGGGCGGAATGGTCTCTTTC | TGGGGACCTTGTCTTCATCAT |
| *FAS* | GGAGGTGGTGATAGCCGGTAT | TGGGTAATCCATAGAGCCCAG |
| *AGPAT1* | GCTGGCTGGCAGGAATCAT | GTCTGAGCCACCTCGGACAT |
| *AGPAT2* | TTTGAGGTCAGCGGACAGAA | AGGATGCTCTGGTGATTAGAGATGA |
| *DGAT1* | GCTGATCCCAGGTTGTTCAT | GAGACAGCTTTGGCCTTGAC |
| *DGAT2* | ACGCAGTCACCCTGAAGAAC | CCCAGGTGTCAGAGGAGAAG |
| *βactin* | GGCTGTATTCCCCTCCATCG | CCAGTTGGTAACAATGCCATGT |

Note: acetyl-CoA acetyltransferase (*ACAT1*); acetyl-CoA carboxylase 1 (*ACC1*); 1-acyl-sn-glycerol-3-phosphate acyltransferase alpha (*AGPAT1*); 1-acyl-sn-glycerol-3-phosphate acyltransferase beta (*AGPAT2*); D-beta-hydroxybutyrate dehydrogenase (BDH1); cytochrome P450-27A1 (*Cyp27A1*); cytochrome P450 cholesterol 7α-hydroxylase (*Cyp7A1*); sterol-12α-hydroxylase (*Cyp8B1*); oxysterol 7α-hydroxylase (*Cyp7B1*); diacylglycerol O-acyltransferase 1 (*DGAT1*); d*iacylglycerol O-acyltransferase 2* (DGAT2); fatty acid synthase (FAS); farnesoid X receptor (*FXR*); hydroxymethylglutaryl-CoA synthase (*HMGCS2*); peroxisome proliferator-activated receptor alpha (*PPARα*); small heterodimer partner (*SHP*); sterol-regulatory element binding protein-1C (*SREBP-1C*); actin beta (*βactin*).

**Table S5.** The topological attributes of co-occurrence networks among different groups

| Topological attributes | PND group | PKD group | AND group | AKD group |
| --- | --- | --- | --- | --- |
| Node | 430 | 282 | 310 | 77 |
| Edge | 2709 | 1104 | 4218 | 439 |
| Average path length | 7.23 | 6.76 | 6.23 | 1.66 |
| Average degree | 12.60 | 7.83 | 27.21 | 11.40 |
| Modularity | 0.79 | 0.76 | 0.55 | 0.29 |
| Clustering coefficient | 0.74 | 0.70 | 0.79 | 0.90 |
| Betweenness centrality | 857.07 | 248.08 | 600.25 | 5.79 |
| Closeness centrality | 0.23 | 0.37 | 0.26 | 0.58 |

**Table S6.** The topological attributes of co-occurrence networks between the FMT-HYK and FMT-HE groups

| Topological attributes | FMT-HE group | FMT-HYK group |
| --- | --- | --- |
| Node | 169 | 415 |
| Edge | 580 | 3984 |
| Average degree | 6.86 | 19.20 |
| Average path length | 2.50 | 6.67 |
| Modularity | 0.87 | 0.64 |
| Clustering coefficient | 0.87 | 0.76 |
| Betweenness centrality | 16.52 | 611.02 |
| Closeness centrality | 0.50 | 0.30 |
